# A Novel Model of Mixed Vascular Dementia Incorporating Hypertension in a Rat Model of Alzheimer’s Disease

**DOI:** 10.3389/fphys.2019.01269

**Published:** 2019-10-24

**Authors:** Paul Denver, Heather D’Adamo, Shuxin Hu, Xiaohong Zuo, Cansheng Zhu, Chihiro Okuma, Peter Kim, Daniel Castro, Mychica R. Jones, Carmen Leal, Marisa Mekkittikul, Elham Ghadishah, Bruce Teter, Harry V. Vinters, Gregory Michael Cole, Sally A. Frautschy

**Affiliations:** ^1^Department of Neurology, David Geffen School of Medicine, University of California, Los Angeles, Los Angeles, CA, United States; ^2^Veterans Affairs Greater Los Angeles Healthcare System, Los Angeles, CA, United States; ^3^Department of Medicine, David Geffen School of Medicine, University of California, Los Angeles, Los Angeles, CA, United States; ^4^Geriatric Research Education and Clinical Center, Greater Los Angeles Veterans Affairs Healthcare System, Los Angeles, CA, United States

**Keywords:** cerebrovascular integrity, tau pathogenesis, Alzheimer disease, vascular dementia, rat model

## Abstract

Alzheimer’s disease (AD) and mixed dementia (MxD) comprise the majority of dementia cases in the growing global aging population. MxD describes the coexistence of AD pathology with vascular pathology, including cerebral small vessel disease (SVD). Cardiovascular disease increases risk for AD and MxD, but mechanistic synergisms between the coexisting pathologies affecting dementia risk, progression and the ultimate clinical manifestations remain elusive. To explore the additive or synergistic interactions between AD and chronic hypertension, we developed a rat model of MxD, produced by breeding APPswe/PS1ΔE9 transgenes into the stroke-prone spontaneously hypertensive rat (SHRSP) background, resulting in the SHRSP/FAD model and three control groups (FAD, SHRSP and non-hypertensive WKY rats, *n* = 8–11, both sexes, 16–18 months of age). After behavioral testing, rats were euthanized, and tissue assessed for vascular, neuroinflammatory and AD pathology. Hypertension was preserved in the SHRSP/FAD cross. Results showed that SHRSP increased FAD-dependent neuroinflammation (microglia and astrocytes) and tau pathology, but plaque pathology changes were subtle, including fewer plaques with compact cores and slightly reduced plaque burden. Evidence for vascular pathology included a change in the distribution of astrocytic end-foot protein aquaporin-4, normally distributed in microvessels, but in SHRSP/FAD rats largely dissociated from vessels, appearing disorganized or redistributed into neuropil. Other evidence of SVD-like pathology included increased collagen IV staining in cerebral vessels and PECAM1 levels. We identified a plasma biomarker in SHRSP/FAD rats that was the only group to show increased Aqp-4 in plasma exosomes. Evidence of neuron damage in SHRSP/FAD rats included increased caspase-cleaved actin, loss of myelin and reduced calbindin staining in neurons. Further, there were mitochondrial deficits specific to SHRSP/FAD, notably the loss of complex II, accompanying FAD-dependent loss of mitochondrial complex I. Cognitive deficits exhibited by FAD rats were not exacerbated by the introduction of the SHRSP phenotype, nor was the hyperactivity phenotype associated with SHRSP altered by the FAD transgene. This novel rat model of MxD, encompassing an amyloidogenic transgene with a hypertensive phenotype, exhibits several features associated with human vascular or “mixed” dementia and may be a useful tool in delineating the pathophysiology of MxD and development of therapeutics.

## Introduction

### The Continuum Between Alzheimer’s Disease, Vascular and Mixed Dementia

The number of people living with dementia globally is projected to reach 81 million by 2040 (Ferri et al., 2005), while cases of Alzheimer’s disease (AD), estimated to be the most prevalent dementia type, are predicted to exceed 13.8 million by 2050 in the United States (Hebert et al., 2013) and 81 million globally (Rizzi et al., 2014; Baumgart et al., 2015), translating to an economic burden associated with dementia of over $800 billion (Wimo et al., 2013, 2017). Despite reports that AD is the most common dementia with an incidence of 50–64%, pure AD is rare as shown in the Religious Orders Study, with a prevalence of only 10%. One reason for this discrepancy is that 60–90% of those diagnosed with Alzheimer’s dementia are found at autopsy to have coexisting cerebrovascular disease (CBVD) and may or may not be diagnosed with mixed dementia (MxD). CBVD pathologies include white matter lesions, microvascular degeneration, microinfarcts and vascular amyloid (Kalaria et al., 2012). Mixed dementia (MxD) refers to the coexistence of vascular pathology with dementia, and the diagnosis depends on the severity of vascular pathology and diagnostic criteria. There may be cortical or subcortical tissue loss, but in all cases of MxD, cerebral small vessel disease (SVD) is evident (Khan et al., 2016; Vinters et al., 2018). Due to the varied criteria for classifying dementia cases as MxD, the incidence is debatable, as reports range widely from 2 to 58%, averaging around 20 to 22% (Jellinger and Attems, 2007; Custodio et al., 2017). Incidence rates at the lower end of this range are usually reported by studies with younger subjects (less prone to MxD) or more rigorous criteria, such as different cutoffs for minimum infarct volume and exclusion of cases with cerebral amyloid angiopathy (CAA) from the criteria.

Critical gaps in knowledge include a paucity of understanding the potential synergism between tau, amyloid and vascular pathology and how this may increase vulnerability to central nervous system (CNS) damage. Vascular pathology in the clinically normal elderly is associated with impaired episodic memory independent of medial temporal atrophy (Wennberg et al., 2019). Furthermore, data from the Honolulu-Asian Aging cohort suggest that the dementia frequency in cognitively normal men more than doubles with increased cerebrovascular lesions accompanied by augmented tau pathology and surprisingly fewer neuritic plaques (Petrovitch et al., 2005), but the manner in which subclinical levels of AD and vascular pathology impacts dementia risk is unclear.

### Cardiovascular Disease Increases Risk for Alzheimer’s Disease and Vascular Dementia

Elevated blood pressure is strongly associated with AD, and mid-life hypertension correlates with greater subsequent cognitive decline (Launer et al., 2000; Petrovitch et al., 2000; Gottesman et al., 2014). Cognitively normal subjects with uncontrolled hypertension show increased amyloid and dementia incidence, particularly in ApoE4 carriers (Rodrigue et al., 2013; Yasar et al., 2013; Oberlin et al., 2015; Walker et al., 2017). Treatment of vascular risk factors may slow decline in AD (Valenti et al., 2014), as suggested by the multi-domain trial in Finnish patients with AD and high CBVD risk (Ngandu et al., 2015). Together these data suggest a meaningful association between cardiovascular health, CBVD, AD and dementia.

Efficacy of drugs directed at pure AD may be attenuated or contraindicated by the presence of CBVD. For example, AD subjects with vascular pathology, such as acute or sub-acute micro-hemorrhage or superficial siderosis, may experience more adverse effects with amyloid-clearing drugs such as anti-amyloid vaccination and may therefore be excluded from clinical trials (Sevigny et al., 2016). Further, a meta-analysis showed memantine to be beneficial after 6 months in moderate to severe AD, but not in patients with mild to moderate vascular dementia (VaD) (Areosa et al., 2005). For these reasons, many AD drug trials deliberately exclude patients with neuroimaging indices of vascular pathology while drug development generally neglects the assessment of efficacy in models of MxD, a major real world patient population.

### Vascular Pathologies and Mechanisms in Mixed Dementia

The molecular mechanisms underlying VaD affect pathogenesis in MxD are unknown. For example, VaD may accelerate AD pathogenesis, including tau and amyloid pathology, by interrupting normal clearance of these proteins from the brain and impeding blood and interstitial fluid flow as a result of vasomotor tone dysregulation (Di Marco et al., 2015; Iadecola, 2016). Alternatively, AD may exacerbate CBVD by contributing to vasoconstriction leading to hypoxia and infarct (Marfany et al., 2018). Some studies suggest that deficient vessel integrity from chronic hypertension decreases cerebral blood flow, leading to hypoperfusion of the brain, protein misfolding and reduced clearance of Aβ and other toxins (de la Torre, 2012). Chronically elevated arterial pressure also leads to thickening of cerebral vessel walls with increased collagen deposition (Zhou et al., 2015), reduced elasticity and narrowing of the lumen (Heagerty et al., 2010). This is especially apparent in small vessels (Feihl et al., 2008; Heagerty et al., 2010), particularly those of the cerebrovasculature (Grinberg and Thal, 2010). The brains of AD patients contain smaller blood vessels, exhibiting reduced density, length and diameter, effects that are especially evident in the vicinity of senile plaques (Bouras et al., 2006; Kitaguchi et al., 2007). Pathologies associated with CBVD include microvascular degeneration, periventricular venous collagenosis and increased vessel tortuosity (Moody et al., 1995; Farkas et al., 2000). Synergistic versus additive effects of CBVD on AD pathogenesis and cognitive decline are not fully understood (Attems and Jellinger, 2014).

### Distinguishing Alzheimer’s Disease From Mixed Dementia

There is no consensus on the neuropsychological parameters that differentiate AD from MxD (Planton et al., 2017; Ramirez-Gomez et al., 2017), although subjects with subcortical ischemic vascular disease, including small vessel disease, commonly show increased difficulty in executive function, but may respond better than AD subjects to cues, suggesting improved preservation of recognition memory, as opposed to AD subjects that have difficulty in coding or storage of memory (Mendez and Ashla-Mendez, 1991; Lafosse et al., 1997; Looi and Sachdev, 1999; Lukatela et al., 2000; Traykov et al., 2002). In addition to impaired executive function, reduced attention, processing speed, and memory retrieval have been reported in MxD (Baker et al., 2012).

Neuroimaging findings can be included in the classification of MxD, but may not necessarily detect mild white matter intensities (WMI) or SVD, another reason the prevalence of MxD may be underestimated (Baker et al., 2012). Additionally, there is no clear consensus on the amount of vascular pathology identified by neuroimaging required to be clinically significant (Dey et al., 2016). Alternatively, the SVD score (0–4), based on white matter hyperintensities, lacunae, microbleeds, and enlarged perivascular spaces detected by magnetic resonance imaging (MRI) is sometimes used to define MxD (Staals et al., 2015).

Although there are NINDS-AIREN and ADDTC criteria for diagnosing MxD, the former including neuropathology and the latter requiring both clinical and neuroimaging abnormalities, there is no clear consensus and no CERAD criteria to define MxD (Jellinger and Attems, 2007). The pathologies of AD and VaD often overlap in subcortical regions (basal ganglia, thalamus, hippocampus and white matter). MxD patients often exhibit multi-infarcts so one definition of MxD requires that patients must meet the criteria for AD and have larger and hemispheral infarcts, reaching 30–50 ml of infarcted volume (Khan et al., 2016), which may indicate synergism between the two pathologies and not just two coexisting pathways. Other groups also include more severe vascular pathology such as territorial infarcts, lobar hematomas, cortical microbleeds or even CAA (De Reuck et al., 2018). It is presumed that in MxD the thresholds for developing dementia may be lowered due to subclinical levels of both pathologies, or that the presence of vascular pathology can unmask AD pathology and trigger dementia (Fischer et al., 1991; Kalaria et al., 2012).

### Models of Cerebrovascular Disease

Models of dementia associated with cerebrovascular insufficiency have typically involved surgical manipulation of the arteries that supply the brain, to invoke transient or chronic hypertensive events (Galisova et al., 2014; Kwon et al., 2014; Wang, 2014; Venkat et al., 2017; Khoshnam et al., 2018; Liu et al., 2018; Yu et al., 2018). Such invasive procedures invariably introduce a multitude of potentially confounding factors and inadequately model the clinical scenario of chronic hypertension. As such, animal models that accurately recreate the clinical scenario of MxD are lacking. Induction of hypertension in a pig model and in 3xTg mice causes shrinkage of hippocampal dendritic arbors, microglial activation, blood brain barrier (BBB) leakage and impaired learning and memory, in addition to elevated amyloid burden (Shih et al., 2018). This suggests that hypertension leads to AD-like brain pathologies in a naïve animal model, while induction of hypertension in an amyloidogenic model can exacerbate pathology further. Hypertension in APP/PS1 mice accelerates progression of AD-like pathologies, including cognitive dysfunction and amyloid pathology, in addition to reduced density of microvessels and cerebrovascular dysfunction (Cifuentes et al., 2015). In naïve APP/PS1 mice, Aβ accumulation in cerebral blood vessel walls has been demonstrated (Klakotskaia et al., 2018). Others have observed microangiopathies and aneurysms throughout the microvessels of the liver, kidneys and the brain of APP/PS1 mice (Kelly et al., 2015, 2017), suggesting a causal relationship between amyloidogenesis and systemic vascular dysfunction, at least in the APP/PS1 mouse. Hypertension in rats results in BBB dysfunction, cognitive impairments and white matter lesions, along with fibrinoid necrosis, hyalinosis and vascular remodeling of small vessels in the brain (Fan et al., 2015). Downregulation of tight junction proteins and disturbed tight junction ultrastructure are apparent in hypertensive rats (Fan et al., 2015; Meissner et al., 2017), and have also been observed in AD and other inflammatory disorders of the brain (Coisne and Engelhardt, 2011; Grammas et al., 2011). Hypertensive cardiovascular disease involves a significant inflammatory component (Harrison et al., 2011), and mounting evidence suggests that inflammation-driven endothelial cell damage leads to BBB breakdown and SVD, an important risk factor in VaD (Wardlaw et al., 2013).

The selectively inbred spontaneous hypertensive rat (SHR) was developed in 1963 at Kyoto University and exhibits elevated systolic blood pressure, cortical and striatal infarcts, along with white matter damage, BBB dysfunction and gliosis that progressively worsens (Tayebati et al., 2012; Kaiser et al., 2014; Venkat et al., 2015). This model also shows evidence of reduced BBB proteins and enhanced permeability within paraventricular and brain stem regions (Biancardi et al., 2014; Meissner et al., 2017) along with progressive elevations of oxidative stress markers in brain and plasma (Takemori et al., 2013). It is widely appreciated that the SHR rat represents an excellent model of essential hypertension leading to cerebral SVD, stroke and VaD (Hainsworth and Markus, 2008; Bailey et al., 2009; Hainsworth et al., 2012; Kaiser et al., 2014). By 6 months of age SHRs develop SVD along with cognitive deficits, hippocampal neurodegeneration and white matter loss (Jalal et al., 2012), detectable by MRI (Koundal et al., 2019). The stroke-prone SHR (SHRSP) was created in 1974, as a sub-strain of the SHR with a high incidence of stroke and hypertension (220–240 mmHg) (Okamoto and Aoki, 1963; Nabika et al., 2012). By 3–4 months of age these rats develop hypertension, which causes elevated plasma levels of pro-inflammatory cytokines (Sicard et al., 2008), and cerebral SVD (Schreiber et al., 2014). In addition to thickened cerebral small vessel walls, these rats also develop cognitive dysfunction, including spatial learning deficits, effects that were ameliorated by COX-2 inhibition (Tang et al., 2015) as well as demyelination and oligodendrocyte apoptosis (Jalal et al., 2012; Weaver et al., 2014), suggesting an association between vascular injury and demyelination, that may be mediated by inflammation.

The SHRSP rats have been reported to have AD like pathology with small but significant increases in hyperphosphorylated tau and Aβ, most notably vascular Aβ associated with the progressive vessel wall damage, thrombotic occlusions and reductions of cerebral blood flow in SHRSP (Bueche et al., 2014; Schreiber et al., 2014; Held et al., 2017; Pirici et al., 2017; Jandke et al., 2018). However, to date there are no animal models of dementia with widespread abundant neuritic plaque pathology and detergent insoluble ptau that incorporate clinically relevant hypertension, making it difficult to investigate the interplay between cerebrovascular damage and ptau and amyloid accumulation.

### Purpose of the Study

This study addresses the need for an adequate model of MxD. Rat models have advantages over mice due to improved cognitive ability and larger volumes of brain, cerebrospinal fluid and plasma, which can expedite development of diagnostic and surrogate imaging and fluid biomarkers. The TgF344-AD rat model, developed by Cohen and colleagues (Cohen et al., 2013), expresses human APP_swe_ and PS1ΔE9 mutations and develops age-dependent cerebral amyloidosis, gliosis and phospho-tau pathology (Cohen et al., 2013). TgF344-AD rats also exhibit pronounced cognitive and neuropsychiatric behavioral abnormalities by 15 months of age, along with dysregulated neural network activity (Munoz-Moreno et al., 2018; Stoiljkovic et al., 2018), as well as age-dependent deterioration of hippocampal synaptic function in this model (Smith and McMahon, 2018). Mouse models harboring amyloidogenic transgenes show hyperphosphorylated tau, but not robust detergent insoluble tau and tangles (tauopathy), neurodegeneration but not robust neuron loss. Introduction of human tau or frontotemporal tau dementia mutations can lead to development of neurofibrillary tangles, but those models do not precisely recapitulate pathogenesis in AD, in part because AD patients do not have tau mutations driving tauopathy (Sydow et al., 2011; Sayed et al., 2018). Here we hypothesized that the introduction of a hypertensive phenotype in an AD rat model could exacerbate Aβ and/or tau pathology, gliosis, vascular pathology and cognitive dysfunction and serve as a new model of MxD for future studies of mechanism, biomarkers and therapeutics.

## Materials and Methods

### Animals

The SHRSP/FAD rat model was developed at the UCLA Division of Laboratory Animal Medicine vivarium, and the colony is now maintained at the Veterans Affairs Greater Los Angeles Health Care System. All experimentation was approved by the UCLA Chancellor’s Animal Research Committee and the Veteran Administration Institutional Animal Care and Use Committee and carried out in compliance with National Institutes of Health guide for the care and use of Laboratory animals (NIH Publications No. 8023, revised 1978). Rats were bred and housed in groups of least two under a 12-h light-dark cycle and had access to standard chow and water *ad libitum.* Four strains were used (16–18 month old, females and males): (i) non-hypertensive WKY (*n* = 8), (ii) TgF344-AD (FAD) (*n* = 11), (iii) hypertensive SHRSP (*n* = 10) and (iv) SHRSP/FAD (*n* = 9) rats. The hypertensive rats in this study were 75:25% SHRSP:F344, and the non-hypertensive rats had 75%:25% WKY:F344 backgrounds, and the methods for breeding them described below.

#### Stroke-Prone Spontaneously Hypertensive Rats With (SHRSP/FAD) or Without (SHRSP) the FAD Transgene

The founder hypertensive rats (SHRSP) were obtained from Charles River Laboratories and the original FAD rats, created at NIH by Dr. Robert Cohen, were obtained directly from his laboratory at Emory as well as purchased from the Rat Resource & Research Center, University of Missouri. The FAD female offspring of the first mating were again crossed with 100% SHRSP males, which produced the SHRSP/FAD litters used in this study. The SHRSP sub-strain of the SHR, created in 1974, is considered a robust model of hypertension and stroke. Although the precise loci are debated, SHRSP genetic susceptibility for hypertension and cerebral lesions is autosomal dominantly inherited (Gratton et al., 1998), allowing us to cross with the TgF344-AD (FAD) rat, producing a novel rat, expressing autosomal dominant familial AD genes, on the SHRSP background (SHRSP/FAD).

The founder FAD rats were derived from the FAD rat on an F344 background, which express human mutant variants of APP (Swedish) and PS1 (ΔE9) and develop age-dependent amyloid pathology, hyperphosphorylation of tau, gliosis and cognitive dysfunction (Cohen et al., 2013). The current hypertensive FAD is 98:2% SHRSP:F344 background.

#### Non-hypertensive Rats With (FAD) or Without (WKY) FAD Transgene

There were two types of non-hypertensive rats (WKY or WKY/FAD). Since the background strain of the SHRSP and FAD rats is WKY and F344, respectively, we bred WKY, the original background of the SHRSP, into the FAD model. Specifically, male WKY rats were paired with female FAD rats. The resulting background was 50:50% WKY/F344, and rats with the FAD transgene were again paired with 100% WKY animals, creating the F2 generation with 75:25% WKY:F344, and the two non-hypertensive groups (FAD and WKY) that were used for the study. The current non-hypertensive FAD colony has a 98% WKY background. The non-hypertensive, non-transgenic control rats are henceforth described as WKY, while the non-hypertensive, transgenic controls are described as FAD rats.

### Blood Pressure Measurement

Arterial blood pressure was measured in the caudal tail artery of rats using the CODA^TM^ Non-invasive Blood Pressure System (Kent Scientific, Torrington, CT, United States). Rats were handled and acclimatized to the apparatus for 15 min daily for 3 days prior to blood pressure measurements. On the fourth day, rats were allowed to enter the holder freely with as little force as possible and allowed to remain in place for 15 min. Then an occlusion cuff was passed over the animal’s tail to the base and inflated to impede blood flow to the tail. The occlusion cuff was slowly deflated, while a second tail cuff that incorporates a volume pressure recording (VPR) sensor was secured to the tail, distal to the occlusion cuff. The tail of the animal was kept in contact with a heated platform, while the VPR sensor measured physiological characteristics of the returning blood flow. As blood returned to the tail, the VPR sensor cuff measured the swelling of the tail that results from arterial pulsations from the blood flow. Systolic blood pressure was automatically measured at the first appearance of tail swelling. Diastolic blood pressure is automatically measured when the increasing rate of swelling ceases in the tail.

### Behavioral Testing

Behavioral testing took place in a quiet, dimly lit room and the testing apparatus was isolated using divider panels. The apparatus was cleaned with 70% isopropanol between trials to avoid the accumulation of olfactory cues. Animals were handled in the testing room for 1 week prior to beginning the experiments, by the experimenter that performed the testing.

#### Open Field Task

Locomotor activity and anxiety were assessed in the open field task (OFT), during which rats were placed into a black box 27.5 inches in width, 27.5 inches in length and with walls 15.5 inches in height. The arena was dimly lit from above, and rats were allowed to explore freely for 8 min. The animals’ movements were tracked with an overhead camera and recorded on Anymaze^TM^ software (Stoelting, Wood Dale, IL, United States). Path length and speed were calculated automatically. An independent investigator, blinded to the identity of the animals, manually counted defecation, rearing and grooming events.

#### Novel Object Recognition Task

The OFT was considered the first habituation day for the novel object recognition task (NOR). The second day of habituation took place 24 h following the OFT. Rats were returned to the same box along with two identical objects, secured to the floor, spaced evenly apart and equidistant from the walls, and allowed to explore freely for 10 min. The objects were either pipette tip boxes or filled cell culture flasks and these were alternated between rats in order to mitigate the effects of preference for or aversion to either object. Twenty-four hours later, rats were again placed into the box with the same identical objects and allowed to explore for 10 min. Rats were then returned to their cage for an interval of 1 h before being placed, once again, into the box with two objects. This time one of the objects was replaced with a previously un-encountered object, either a tip box or a flask, and the rat was, again, allowed to explore freely for 10 min. The time spent exploring each object was measured and recognition indices calculated for each object, which were then compared to determine whether or not the animal showed a significant preference for the novel object, an innate behavior in healthy rats. A discrimination index (DI) was also calculated for each mouse in the test phase with the equation (tN – tF)/(tN + tF), where tN equals time spent exploring the novel object and tF equals time spent exploring the familiar object. Another measurement of preference was, the recognition index (RI) calculated by tN/(tN+tF).

#### Y Maze

The Y maze consisted of three arms and walls 12 inches in height. Testing took place another day following completion of the NOR task. The rat was placed into the bottom arm of the Y maze, designated A and allowed to explore the rest of the maze freely. The animals’ movements were tracked with an overhead camera and recorded on Anymaze software, measuring entries into each arm (A, B, and C). We then calculated corrected arm entries as those into a “novel” subsequent arm (e.g., A > B > C or B > C > A or C > B > A). Errors were measured as entries into a directly previous arm (e.g., A > B > A or C > B > C or B > A > B). Spontaneous alternation was calculated with the equation C/(T-1) × 100, where C equals correct arm entries and T equals total arm entries.

### Euthanasia, Plasma and Brain Collection for Biochemistry and Histology

At the end of the experiment, rats were injected with a lethal dose of pentobarbital (100 mg/kg i.p.) and upon deep anesthesia, the chest cavity was opened and rats were perfused intracardially with a physiologically isotonic buffer containing 10 mM HEPES, 137 mM NaCl, 4.6 mM KCL, 1.1 mM KH2PO4, 0.6 MgSO4, 1.1 mM EDTA, and protease inhibitors (5 mg/ml of leupeptin and aprotinin and 2 mg/ml pepstatin A, pH 7.4). The brain was bisected and the hippocampus and cortex were dissected from the left hemisphere, snap frozen in liquid nitrogen and stored at −80°C until use for biochemistry, while the right hemisphere was immersion-fixed in 4% formalin, sucrose cryopreserved and frozen at −80°C until cryosectioning.

### Histology

#### Immunohistochemistry

Coronal sections of frozen rat hemi-brains were cryosectioned at 12 μm thick, mounted on slides and stored at −20°C. For immunohistochemistry (IHC), slides were warmed to room temperature for 1 h and then steamed for 20 min using a citric acid base antigen-unmasking solution (Vector Labs, Burlingame, CA, United States). Sections were quenched with hydrogen peroxide (H_2_O_2_) in methanol for 30 min at room temperature, then washed three times with tris buffered saline (TBS) (pH 7.4) (for staining of Aβ, Tau pS422, collagen-4) or treated with 0.3% Triton X-100 in 0.1 M TBS (pH 7.4) for 10 min at room temperature (for staining of Aqp-4, GFAP, Tau pS422, collagen-IV). For Aβ staining, sections were pretreated with 70% formic acid for 10 min at room temperature. For all IHC, sections were treated with a blocking solution, consisting of 1.5–5% normal serum and 3% bovine serum albumin (BSA) in TBS for 1 h at 37°C, followed by incubation with primary antibodies for 1 h at 37°C, then overnight at 4°C. Amyloid-β (Aβ) deposits were labeled with 6E10 (1:500, anti-Aβ) recognizing residues 1-16 (Biolegend, San Diego, CA, United States). Astrocytic endfeet associated with capillaries were labeled with anti-Aqp-4 polyclonal (1:200, NOVUS Biologicals, Littleton, CO, United States) and processes with anti-GFAP monoclonal (1:5000; Sigma-Aldrich St. Louis, MO, United States). Anti-calbindin D 28K rabbit polyclonal antibody (1:800; Thermo Fisher, Asheville, NC, United States) was used as a neuronal marker. Microglia were labeled with an anti-Iba1 rabbit polyclonal (1:200; Wako, Richmond, VA, United States) antibody raised against a synthetic peptide corresponding to the C-terminus of Iba1. Levels of Tau phosphorylated at serine 422 (pS422) were detected using a rabbit polyclonal antibody against MAPT/Tau pS422-Aff-purified (1:800, Acris, San Diego, CA, United States). Blood vessels were labeled with anti-collagen IV mouse monoclonal antibody (Invitrogen, Waltham, MA, United States). Sections were then incubated with secondary antibodies diluted in normal serum and 3% BSA for 1 h at 37°C followed by avidin-biotin complex (ABC; Vector Labs, Burlingame, CA, United States) reagent for 1 h 20 min at 37°C. After ABC incubation, metal-enhanced peroxidase diaminobenzidine (DAB; Pierce, Rockford, IL, United States) was used for detection of positive staining.

#### Luxol Fast Blue Myelin Staining

The Kluver-Barrera Luxol Fast Blue Method was used to stain myelin using cryostat sections. Briefly, sections were rinsed in 95% alcohol, then incubated overnight at 60°C with a 0.1% solution of Luxol Fast Blue (Solvent Blue 38 Sigma) dissolved in 95% alcohol, 0.5% and ∼10% glacial Acetic Acid, then filtered. Sections were washed with 95% ethanol, rinsed with distilled water, then differentiated by quick immersion in 0.05% lithium carbonate solution, followed by several changes in 70% ethanol and then rinsed with distilled water. Then sections are incubated for 6 min with 60°C filtered 0.1% Cresyl Echt Violet solution in distilled water with 15 drops of 15% glacial acetic acid added. Sections were differentiated and acidified (drops of 5N HCL) in 95% alcohol then cleared in CitriSolv^TM^ Solvent and Clearing Agent (VWR) prior to cover slipping with Thermo Scientific^TM^ DPX Mounting Media. Evaluation of differences in Luxol Blue staining patterns was performed by an experimenter blinded to transgenic animal. Four consecutive coronal sections were evaluated per rat at Bregma at −4.0.

### Western Blot

Brain tissue was weighed before starting protein extraction. Frozen brain tissues were added to TBS (10 × volume of brain wet weight) containing complete protease inhibitor (PI) and phosphatase inhibitor (PPI) cocktail (Roche, Mannheim, DE, United States) and sonicated for 10 s × 3 times on ice to disrupt cell membranes. The sonication conditions were kept the same in all the following steps. After sonication, samples were centrifuged at 132,000 × *g* for 20 min at 4°C to produce TBS supernatant used to measure soluble proteins. The pellet was further extracted in modified RIPA lysis buffer with PI and PPI to obtain detergent-soluble membrane fractions and lysis-insoluble pellets for insoluble tau aggregates. Lysis pellets were then re-suspended in sample buffer. All the samples were stored at −80°C and protein assayed prior to use. The protein concentration was measured according to Bio-Rad protocol (Bio-Rad, Hercules, CA, United States). For western blotting, 15–30 mg of protein was electrophoresed on a 7.5–12% acrylamide gradient gel, and then transferred to polyvinylidene fluoride membrane. After blocking in 10% non-fat milk, blots were incubated with primary antibodies against Aqp-4 (NOVUS Biologicals, Littleton, CO, United States), GFAP (Sigma-Aldrich, St. Louis, MO, United States), tau pS202 (CP13; gift from Peter Davies, Albert Einstein College, New York), caspase-cleaved actin (fractin from author Greg Cole, Yang et al., 1998), platelet-endothelial cell adhesion molecule-1 (PECAM-1), Synaptosome Associated Protein 25 (SNAP25), synaptophysin, drebrin and *N*-methyl D-aspartate receptor subtype 2B (NMDAR2B or NR2B), NR2B (Santa Cruz Biotechnology) then incubated with horseradish peroxidase-conjugated secondary antibodies. SuperSignal West Femto Substrate (Pierce, Rockford, IL, United States) was exposed on x-ray film below saturation, and bands were scanned and quantified using a UVP bio-imaging system (UVP, Upland, CA, United States).

### Mitochondrial Complexes

Mitochondrial enzymes were measured by western blot, using the premixed cocktail of primary monoclonal antibodies provided in the OxPhos Panel kit (Abcam, previously MitoSciences) ab110413) against Complex I subunit NDUFB8 (ab110242), CII complex II-30kDa (ab14714), Complex III-Core protein 2 (ab14745) Complex IV subunit I (ab14705) and Complex 5V alpha subunit (ab14748). The lysis fraction from the hippocampal extract was used, and samples loaded as described above.

### Plasma Exosomes

Plasma was collected at euthanasia, prior to perfusion and stored at −80°C. Plasma exosomes were isolated according to the method of exosome precipitation (Fiandaca et al., 2015). Briefly, 250 μl of plasma was spun at 3000 × *g* for 15 min then the supernatant with added protease inhibitor cocktail (Roche Applied Sciences, Inc.) was incubated with 100 μl thromboplastin-D (Thermo Scientific, Inc.) at room temperature for 60 min. After spinning at 13,500 rpm for 5 min, the supernatant with added protease inhibitor cocktail was mixed with 63 μl of ExoQuick^TM^-TC exosome precipitation solution (System Biosciences, Inc.) and incubated at 4°C for 60 min. The samples were again spun at 1500 × *g* for 30 min, were then removed and the supernatant was spun again for 5 min. The pellets were re-suspended in 1 × PBS with H_2_O/protein inhibitor. Then the isolated exosomes were purified by Exo-spin^TM^ column (Cell Guidance Systems, Inc.) according to the manufacturer’s instructions. After purification, exosomes were run on reduced 6–15% Tris-Glycine/SDS gel and western blot was run using an anti-Aqp-4 (Novus Biologicals, Littleton, CO, United States) antibody.

### Statistical Analysis

#### Western and Mitochondrial Analysis

Statistical analysis was performed using SPSS V.22 (IBM, Inc). Two way ANOVA [SHR (WKY or SHRSP) × FAD (Tg- or Tg+)] was performed to determine differences in protein levels, and where applicable, Multivariate Analysis (MANOVA) was performed. Log or square root transformation for data with unequal variances. Two-way ANOVA followed by *post hoc* LSD or *t-*tests was used to analyze behavioral data. *P*-values lower than 0.05 were considered statistically significant. *P*-values between 0.1 and 0.05 were considered trends.

#### Immunohistochemical Analysis

Data for immunohistochemistry analysis were collected from microscopic images acquired using a Macintosh computer with a digital MC170 5 MPixel Leica camera on an Olympus Vannox-T (AHBT) microscope. Images were then analyzed using the public domain software Image J, http://rsbweb.nih.gov/ij/. Immunohistochemical data were analyzed quantitatively using two-way ANOVA (FAD × SHRSP × brain region), and differences between strains were determined by Fisher’s least significant difference (LSD) *post hoc* analysis. Sex was included in the model only if initial analysis showed significant effects (*p* < 0.05). A sex effect was identified only for Aqp-4 western blot, as females showed the highest Aqp-4 increases in SHRSP/FAD. Four consecutive sections and the bregma specific for the region of interest were assessed per rat. Density thresholding was used with Image J macros to evaluate integrative density, size of individual cells number of cells per area, and percentage area stained.

## Results

### SHRSP/FAD Rats Show Hypertension and Preservation of the SHRSP Phenotype

**FIGURE 1:**
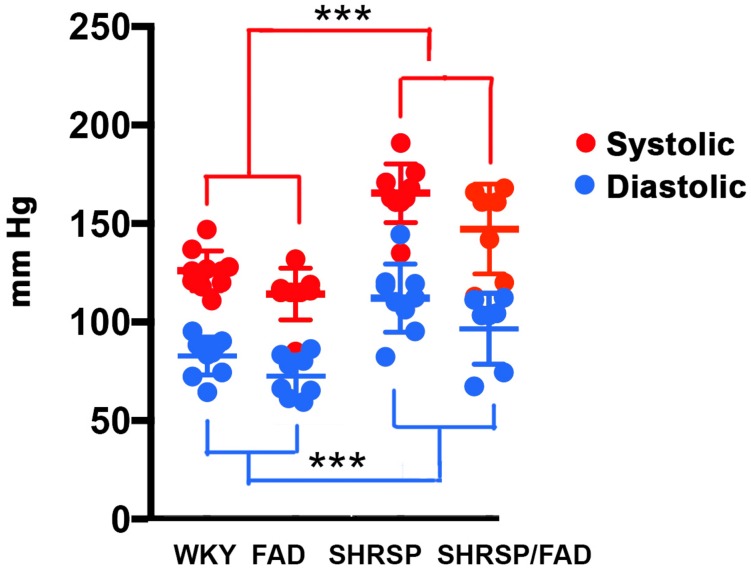
Systolic and diastolic blood pressure is elevated in SHRSP and SHRSP/FAD rats. Shown are systolic (red) and diastolic (blue) blood pressure measurements in FAD (*n* = 11), SHRSP (*n* = 10), and SHRSP/FAD (*n* = 9) rats, as well as non-transgenic WKY controls (*n* = 8), as measured in the rats’ caudal tail artery. The multivariate analysis showed a significant effect of SHRSP only (*p* < 0.0001), indicating that SHRSP but not sex or FAD transgene influenced blood pressure. Similarly the univariate *F* tests showed significant main effects of SHRSP on both diastolic and systolic pressure (*p* < 0.0001 and *p* < 0.0001, respectively). The univariate effect of FAD on diastolic and systolic pressure was significant (*p* < 0.019, respectively), but *post hoc* analysis showed that the trend for slightly higher pressure in SHRSP/FAD rats was marginal and not significant. *Post hoc* analysis showed that both systolic and diastolic pressure were higher in SHRSP and SHRSP/FAD rats than in WKY and FAD (*p* < 0.001), consistent with preservation of the SHRSP hypertensive phenotype in the SHRSP/FAD rat. Data represent means ± SEM. ^∗∗∗^*p* < 0.001; MANOVA with Pillai’s Trace. Log transformation of data was performed to establish homogeneity of variance.

Blood pressure readings were acquired from the caudal tail arteries of the four strains (Figure 1). Data were analyzed via MANOVA [variables (diastolic and systolic) × SHRSP × FAD × sex], and log transformation of data was needed to establish equal variance. The multivariate main effect was only significant for SHRSP [Pillai’s Trace = 0.719, *F*(4,50), *p* < 0.0001] as was the univariate main effect for SHRSP, on diastolic and systolic pressure [*F*(2,33) = 11.565, *p* < 0.0001 and *F*(2,33) = 21.044, *p* < 0.0001, respectively]. Although univariate tests revealed a main effect of FAD, *post hoc* analysis showed only incremental (5%) non-significant increases in blood pressure in FAD rats, compared to WKY. In contrast, *post hoc* analysis showed that both systolic and diastolic pressures were higher in SHRSP and SHRSP/FAD rats, compared to FAD and WKY rats (*p* < 0.001), consistent with preservation of the SHRSP hypertensive phenotype in this new SHRSP/FAD model.

### Amyloid and Tau Pathology in the Hippocampus

#### Amyloid-β Plaque Burden in SHRSP/FAD Is Slightly Reduced and Plaques Have More Diffuse Morphology Than in FAD Rats

**FIGURE 2:**
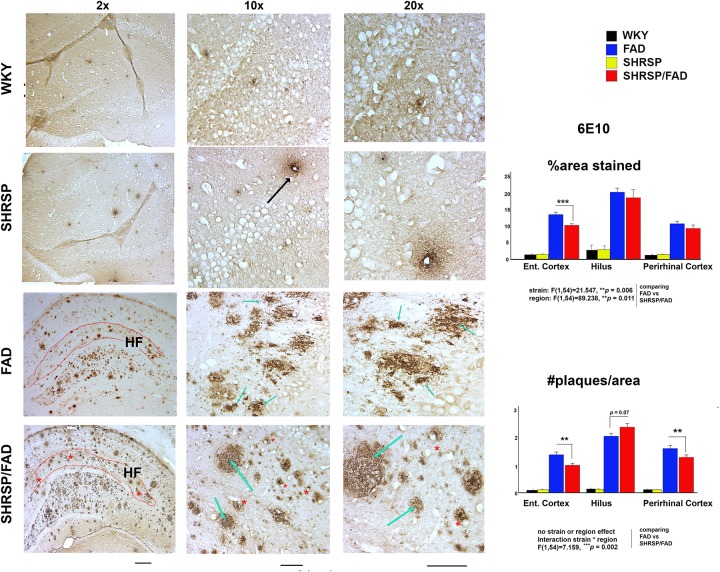
Neuritic Amyloid-β plaques in FAD rats become more diffuse with hypertension. Micrographs depicting Aβ (6E10) immunohistochemical staining of brain sections (hippocampus) from FAD (*n* = 11) and SHRSP/FAD (*n* = 9) rats are shown. No plaques were observed in WKY rats, while there was sparse vascular staining in the SHRSP rat (black arrow), unlike advance plaque pathology in the FAD and SHRSP/FAD groups. With the low magnification micrographs, more compact, neuritic plaques (black arrows) were observed in FAD rats, whereas plaques in the hippocampus of SHRSP/FAD rats rarely had compact cores and were more diffuse (blue arrows) and frequently associated with vessels (red asterisks). The highly vascularized hippocampal fissure is outlined in red. Although there was moderate staining for Aβ in the HF of FAD rats, the SHRSP/FAD rats showed extensive staining of plaques and vessels (red asterisks). Since there was negligible staining in the SHRSP and WKY groups, quantitative analysis was performed in the plaque-rich hilus, measuring Aβ plaque number and average percentage area only in the FAD and SHRSP/FAD groups. Two-way ANOVA (region × strain) of percent area and count, showed significant region effects, which *post hoc* analysis revealed was due to higher plaque burden and count in the hilus. For percent area, there was also a significant main effect of strain and *post hoc* analysis showed that this was due to a reduction in plaques in the entorhinal cortex of SHRSP rats. For plaque count, two-way ANOVA showed an interaction between strain and region. *Post hoc* analysis showed significant reductions in plaque number in both the entorhinal and perirhinal cortices of SHRSP rats. Data represent means ± SEM. ^∗^*p* < 0.05, ^∗∗^*p* < 0.01; two-way ANOVA. Log transformation of data for percent area was also performed to establish homogeneity of variance.

Micrographs in Figure 2 depict Aβ deposition (6E10) in the hippocampus of FAD and SHRSP/FAD rats, while no plaques were observed in WKY rats. Quantitative analysis of plaque number and percentage area stained was assessed in three brain regions, the hilus (highest density of plaques), perirhinal and entorhinal cortices (moderate plaque burden). Two-way ANOVA was performed (strain × region) and log transformation was used to obtain equal variances for percentage area stained. The main effects for strain and region were significant (*p* = 0.006 and *p* = 0.011, respectively) and while there was no interaction between strain and region, *post hoc* analysis revealed a reduction in plaque numbers in the entorhinal cortex of SHRSP/FAD rats compared to FAD in (*p* = 0.001). Two-way ANOVA of plaque number showed that the effects of strain or region were non-significant, but there was a significant interaction between strain and region (*p* = 0.002).

*Post hoc* analysis revealed that Aβ plaque number was significantly reduced in the entorhinal and perirhinal cortices of SHRSP/FAD rats (*p* < 0.05), whereas plaque number was increased, albeit non-significantly (*p* = 0.07), in the hilus of SHRSP/FAD rats, compared with FAD. Furthermore, plaques in the SHRSP/FAD rats were more diffuse, when compared with those in FAD rats, which were of coarser texture, typical of neuritic plaques.

#### Tau (pS422) Staining of Neurites and Cell Bodies and Insoluble Tau (CP13) Levels Are Increased in SHRSP/FAD Rats

**FIGURE 3:**
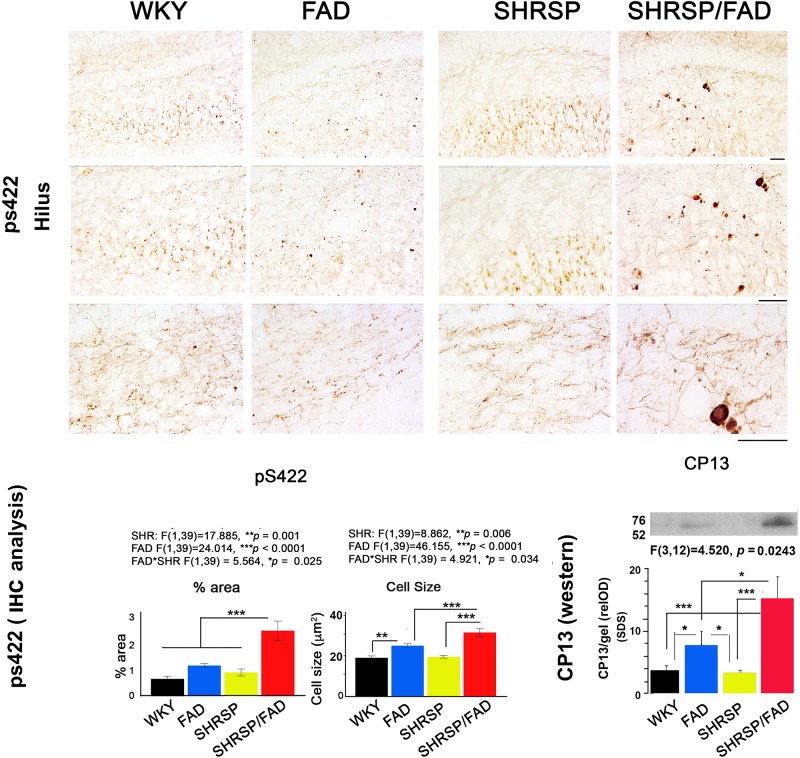
Tau staining and insoluble protein levels are increased in SHRSP/FAD rats. Micrographs depict positive tau pS422 staining in the hilus of the hippocampus of WKY (*n* = 8), FAD (*n* = 11), SHRSP (*n* = 10), and SHRSP/FAD (*n* = 9) rats. Only SHRSP/FAD rats showed intense staining of neurons labeling globose neurofibrillary tangle-like structures extending tortuous neurites. Two-way ANOVA of percent area and cell size showed significant main effects of SHRSP (*p* = 0.001, *p* = 0.006), FAD (*p* = 0.0001, *p* = 0.0001) and SHRSP × FAD interactions (*p* = 0.025, *p* = 0.034), respectively. Percentage area stained positive for tau pS422 was increased more than two-fold in the SHRSP/FAD rats, compared to WKY, FAD and SHRSP rats (*p* < 0.0001). *Post hoc* analysis indicated that cell size was significantly elevated in the hilus of FAD rats, compared to WKY (*p* < 0.01), while further elevations of tau pS422 cell size were detected in SHRSP/FAD rats, compared to FAD and SHRSP rats (*p* < 0.001). The lower right panel shows representative lanes from a western blot analysis of CP13, an antibody to the serine 202 phospho-epitope of tau from the insoluble SDS fractions of hippocampal lysates. Two-way ANOVA showed a significant FAD effect and a FAD × SHR interaction. *Post hoc* analysis showed that there was a non-significant trend for increased CP13 levels in hippocampus from FAD compared to WKY rats (*p* = 0.08). However, in SHRSP/FAD rats, levels were significantly elevated compared to SHRSP rats (*p* < 0.001). Furthermore, CP13 levels were significantly elevated in the hippocampus of SHRSP/FAD rats, compared to FAD (*p* < 0.05). Data represent means ± SEM. ^∗^*p* < 0.05, ^∗∗^*p* < 0.01, ^∗∗∗^*p* < 0.001; two-way ANOVA with LSD Fisher’s *post hoc* test.

In Figure 3, micrographs depict Tau pS422 staining in the hippocampal hilus of the four strains. Unlike WKY, SHRSP and FAD rats, which showed light staining of their hilar neurons, SHRSP/FAD rats showed intense staining of occasional neurons with pre-tangle morphology, similar to globose neurofibrillary tangles, and a few displaying tortuous neurites. Two-way ANOVA analysis of both percentage area stained and cell size, showed significant main effects of SHRSP (*p* = 0.001 and *p* = 0.006), FAD (*p* < 0.0001 and *p* < 0.0001) and SHRSP × FAD interactions (*p* = 0.025 and *p* = 0.034). For percentage area stained, the SHRSP/FAD rats showed two-fold increases in levels above all other strains (*p* < 0.0001), which contributed to the interaction between SHRSP and FAD. Despite the significant main effect of FAD on percentage area of tau pS422 staining and incremental increases in FAD and SHRSP rats, compared to WKY, these differences were not significant with *post hoc* analysis. In contrast, *post hoc* analysis showed that tau pS422-positive cell size was significantly elevated in FAD rats, compared to WKY (*p* < 0.01) and in SHRSP/FAD rats, compared to FAD (*p* < 0.001) and SHRSP rats (*p* < 0.001). Together, this suggests that tau pS422 staining is robustly increased in the hilus of SHRSP/FAD rats, compared to FAD or SHRSP alone.

The lower right panel of Figure 3 shows representative lanes from western blot experiments measuring tau protein (CP13 antibody) in the detergent lysis buffer insoluble pellets extracted with SDS from hippocampi from the four rat strains. Two-way ANOVA showed significant main effects for FAD effects and the FAD × SHRSP interaction. *Post hoc* analysis showed a non-significant trend for increased detergent insoluble CP13 levels in FAD, compared to WKY rats. Whereas levels of CP13 in the hippocampus were significantly elevated in SHRSP/FAD rats, compared to WKY (*p* < 0.001), FAD (*p* < 0.05), and SHRSP rats (*p* < 0.001). These data suggest that the small increase of insoluble tau levels in the hippocampus of FAD rats is robustly increased in the SHRSP/FAD rat.

### Neuroinflammation

#### Iba1

**FIGURE 4:**
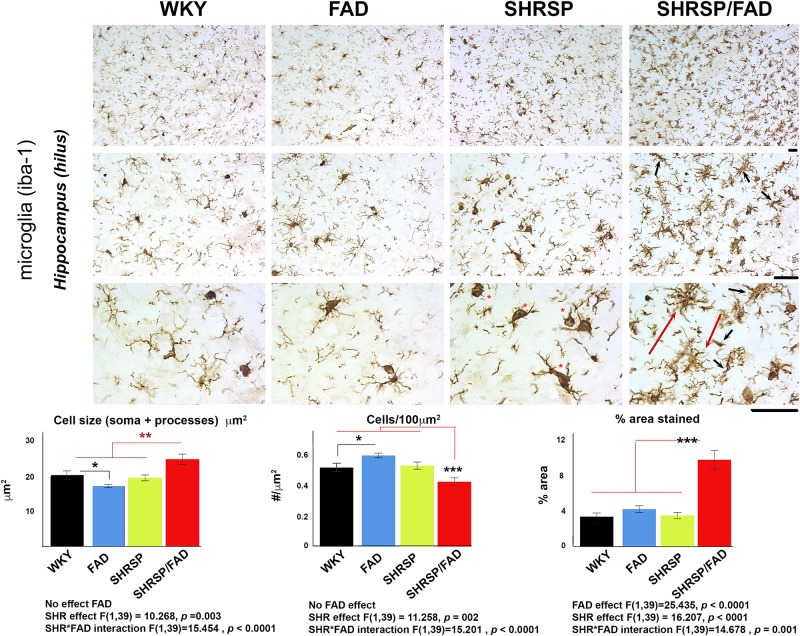
Microglial staining and cell size are increased in SHRSP/FAD rats. Micrographs show Iba1-positive microglia in the hippocampus of the four strains. Microglia in SHRSP, FAD and WKY rats all showed normal ramified morphology with well-defined soma, which was more pronounced in the SHRSP rats (red asterisks). In contrast, microglia in the SHRSP/FAD rat showed hyper-ramified, thickened bushy processes (red arrows) and narrowed soma, some with rod morphology (black arrows). Two-way ANOVA (FAD × SHRSP) and showed a main FAD effect in percent area (*p* < 0.0001) and a significant SHRSP × FAD interaction (*p* = 0.001). *Post hoc* analysis showed that percent area stained for Iba1 was elevated in the hippocampus of SHRSP/FAD rats, compared to all other groups (*p* < 0.001). In fact this interaction was also seen with cell number and cell size, which *post hoc* analysis revealed was similarly due to significantly increased Iba1-positive cell number in SHRSP/FAD rats, compared to all other groups combined (*p* = 0.005). Number of microglia was significantly increased in FAD rats, compared to WKY, whereas cell count was significantly reduced in SHRSP/FAD rats, compared to SHRSP, despite increased area, associated with processes in SHRSP/FAD animals. Data represent means ± SEM. ^∗^*p* < 0.05, ^∗∗^*p* < 0.01, and ^∗∗∗^*p* < 0.001; two-way ANOVA with LSD Fisher’s *post hoc* test.

Illustrated in Figure 4 are micrographs showing microglial staining in the hilus of the hippocampus of the four strains. Brains of WKY, FAD and SHRSP rats displayed microglia with reactive ramified processes and well-defined, darkly stained oval soma, which was most pronounced in SHRSP rats (red asterisks). In contrast, in the SHRSP/FAD group, microglia were hyper-ramified with thickened bushy processes (red dashed arrows) and narrowed soma, some with occasional rod morphology (black arrows). Total percentage area stained, cell size (soma and branches) and cells per unit area were evaluated using two-way ANOVA (FAD × SHRSP). There were no significant FAD effects on Iba1-positive cell size or count, but there was a main FAD effect (*p* < 0.0001) on percentage area stained and a significant SHRSP × FAD interaction (*p* = 0.001). *Post hoc* analysis showed that the significant SHRSP × FAD interaction was related to an elevation of percentage area of Iba1 staining in the hippocampus of SHRSP/FAD rats, compared to all the other groups (*p* < 0.001). In fact, this significant interaction was also seen with cell count and size, which *post hoc* analysis revealed was similarly due to changes in the SHRSP/FAD rats but not the FAD group. *Post hoc* analysis also demonstrated a small FAD effect associated with increased microglia in FAD rats (*p* < 0.05), but in SHRSP/FAD rats, the FAD effect was associated with fewer microglia (*p* < 0.001), despite increased percentage area stained.

#### Hypertrophic Astrocytes and Disorganized Aquaporin-4+ End-Feet in SHRSP/FAD Rats

**FIGURE 5:**
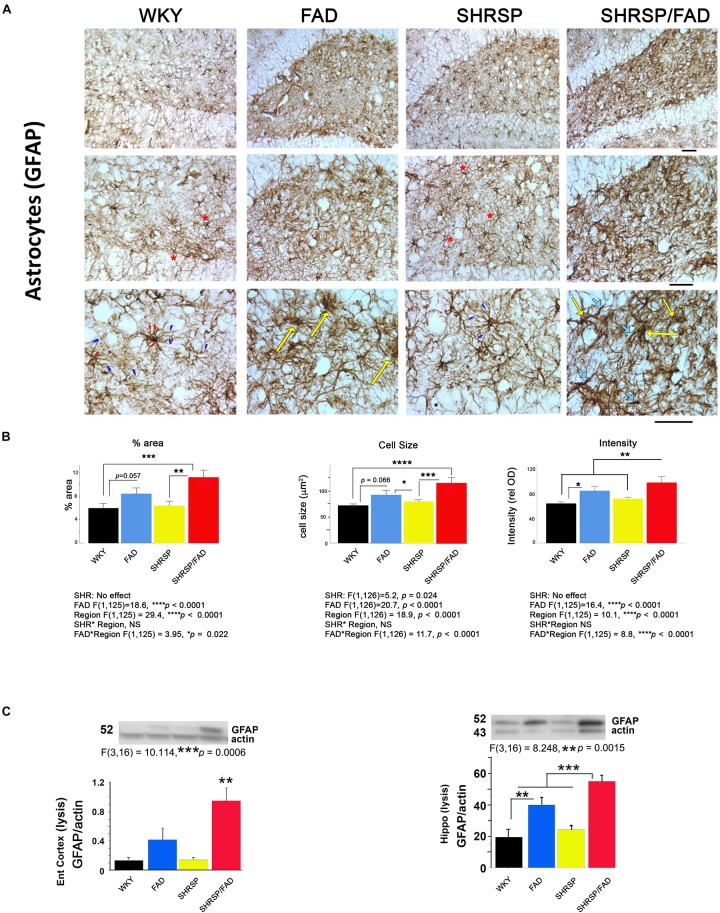
Astrogliosis is augmented in SHRSP/FAD rats. **(A)** Micrographs show immunostaining of brain sections for the astrocytic antigen GFAP. In WKY rats, soma generally extended thick primary branches (red arrowheads), from which multiple secondary and tertiary processes (blue arrowheads) diverged with star-shaped soma typical (red asterisks) of quiescent astrocytes. There was increased GFAP staining throughout the hilus (similar to other regions, not shown) in SHRSP/FAD rats, manifested by hypertrophied soma (yellow arrows) and extended thickened and dense processes (thick light blue arrows) with fewer thin, secondary and tertiary processes, consistent with an activated state. **(B)** Quantitative image analysis showed that, percentage area of GFAP staining was significantly elevated in SHRSP (*p* = 0.057) and SHRSP/FAD (*p* = 0.001) rats when compared with WKY, and in SHRSP/FAD rats when compared with FAD (*p* = 0.005). Two-way ANOVA (region × SHRSP × FAD) revealed significant main effects for all variables (*p* < 0.0001), due to a robust increase in GFAP staining in the hilus of FAD rats relative to plaque-sparse regions. GFAP staining intensity was increased further in the hilus of SHRSP/FAD rats (*p* < 0.006). In SHRSP/FAD rats, however, all variables, including cell size, percentage area stained and GFAP staining intensity, were significantly elevated, compared to WKY and SHRSP rats. **(C)** The lower two panels show representative western blot gels from experiments measuring GFAP protein in the lysis fraction of tissue lysates from the entorhinal cortex and hippocampus. One-way ANOVA showed a significant effect of strain on GFAP protein levels in the entorhinal cortex (*p* = 0.006) and hippocampus (*p* = 0.0015) and *post hoc* analysis indicated that GFAP protein levels were significantly elevated in entorhinal cortex and hippocampal tissue from SHRSP/FAD rats, compared to all other groups. GFAP was also significantly elevated in the hippocampus of FAD rats, compared to WKY. Data represent means ± SEM. ^∗^*p* < 0.05, ^∗∗^*p* < 0.01, ^∗∗∗^*p* < 0.001

Figure 5 shows immunostaining of brain sections for the astrocytic antigen GFAP. In Tg- WKY control rats, soma generally extended thick primary branches, from which multiple secondary and tertiary processes diverged. There are many star-shaped soma typical of quiescent astrocytes. The most noticeable difference between strains was increased GFAP staining throughout the hilus and other regions of the brain (not shown) in SHRSP/FAD rats. These SHRSP/FAD rats manifested hypertrophied soma size, and extended thickened and dense processes with fewer thin, secondary and tertiary processes, consistent with immune activation. There was also a similar but more modest effect in FAD rats as well as an SHRSP effect. Specifically, astrocytes in the SHRSP rats had fewer processes but more cells than WKY, consistent with SHRSP-dependent proliferation. Data collected from image analysis were analyzed by two-way ANOVA (region × SHRSP × FAD), which revealed significant main effects. Data from square root transformation of percentage area stained was used for ANOVA to establish equal variance. There was a significant main effect of the FAD transgene on percentage area stained, cell size and GFAP staining intensity (*p* < 0.0001). *Post hoc* analysis indicated that while the trends for increased percentage area of GFAP staining (*p* = 0.057) and cell size (*p* = 0.066) in FAD, compared to WKY did not reach significance, percentage area stained (*p* < 0.001) and cell size (*p* < 0.0001) were robustly and significantly elevated in the hippocampal hilus of SHRSP/FAD rats, compared to WKY. Additionally, staining intensity was significantly elevated in SHRSP/FAD rats, compared to FAD (*p* < 0.006). Furthermore, *post hoc* analysis showed that percentage area (*p* = 0.005) and cell size (*p* < 0.001) were elevated in the dentate gyrus of SHRSP/FAD rats, compared to SHRSP. There were no SHRSP interactions, only a main SHRSP effect on cell size (*p* = 0.024), corresponding to a significant reduction of cell size in SHRSP, compared to FAD rats (*p* < 0.05). Percentage area stained, cell size and GFAP staining intensity all showed a strong region effect (*p* < 0.0001) corresponding to more robust increases in the plaque-enriched hilus of FAD and SHRSP/FAD rats.

#### GFAP Protein Levels in the Entorhinal Cortex and Hippocampus Are Increased in SHRSP/FAD Rats

The bottom panel of Figure 5 depicts representative lanes of western blots for GFAP in two brain regions and the densitometric analysis of bands normalized to actin. Consistent with observations by IHC, one-way ANOVA showed that there was a main effect of strain (*p* < 0.001). *Post hoc* analysis showed that levels of GFAP were significantly elevated in the lysis fraction from the entorhinal cortex and hippocampus of FAD rats, compared with WKY. However, in SHRSP/FAD rats, levels in both the entorhinal cortex and hippocampus were higher than in all other groups. Together, these data are consistent with multiple brain regions showing increased astrogliosis in FAD rats, which is exacerbated in SHRSP/FAD animals.

#### Aquaporin-4 Staining of Astrocyte End-Feet in the Hippocampus Indicates Disruption of Morphology in SHRSP/FAD Rats Along With Overall Increased Level of Staining

**FIGURE 6:**
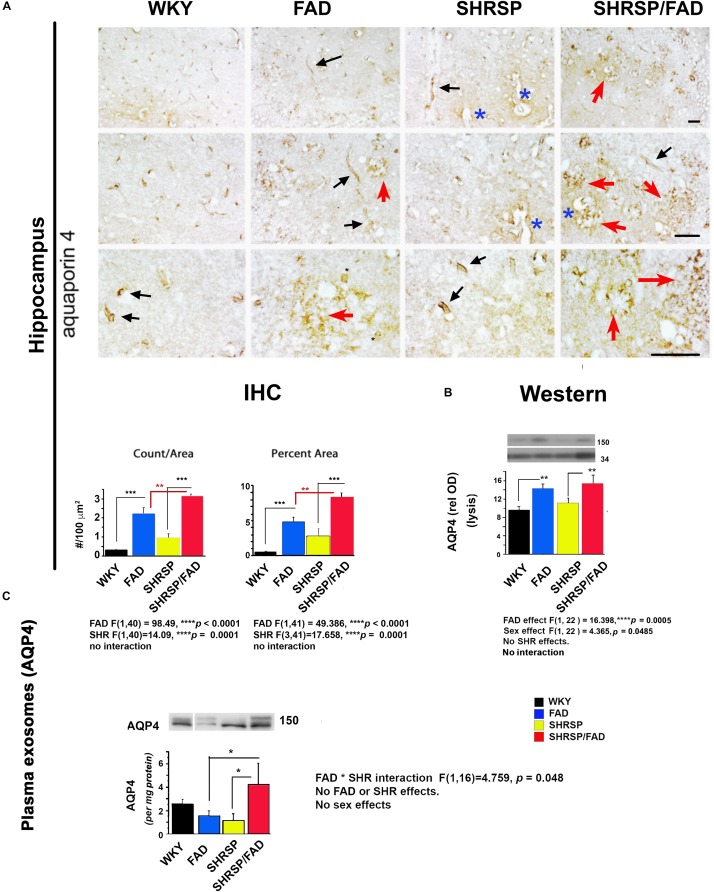
Aquaporin-4 is elevated in the brains of FAD, with a further increase in SHRSP/FAD rats, while aquaporin-4 protein levels in plasma exosomes were elevated in SHRSP/FAD rats only. **(A)** Micrographs illustrate staining for Aqp-4, a membrane-bound protein in astrocyte end-feet. In WKY rats, staining was associated with evenly distributed small tubular vessels (black arrows), while in FAD rats, staining was in patches of fragmented vessels, tortuous thin astrocyte processes (red arrows) and swollen vessels (blue asterisks). In SHRSP rats, Aqp-4 staining was slightly increased and labeled distended but normally shaped tubular vessels (black arrows). There were more Aqp-4-positive amorphous patches in the hippocampus of FAD rats, an effect that was exacerbated in SHRSP/FAD rats (red arrows). Two-way ANOVA showed FAD (*p* < 0.0001) and SHRSP (*p* < 0.0001) effects for count and percent area. *Post hoc* analysis showed that in the hippocampus, Aqp-4 count (*p* < 0.001) and percentage area stained (*p* < 0.001) were significantly elevated in FAD, compared to WKY rats. This effect was further increased in SHRSP/FAD rats in which Aqp-4 count (*p* < 0.01) and percentage area stained (*p* < 0.01) were significantly increased, compared to FAD rats. In SHRSP/FAD rats, there were more structures stained positively for Aqp-4 (*p* < 0.001) and a higher percentage area stained (*p* < 0.001), compared to SHRSP. **(B)** Aqp-4 was also measured in hippocampal tissue lysates (mid right panel) and analyzed by two-way ANOVA (FAD × SHRSP × sex), which showed a significant main effect of FAD on the 150 kDa Aqp-4. *Post hoc* analysis demonstrated that Aqp-4 (150 kDa) was significantly elevated in the hippocampal tissue from FAD, compared to WKY and SHRSP rats (*p* < 0.01). **(C)** The 150 kDa Aqp-4 protein was also measured in brain-derived plasma exosomes (bottom panel) and two-way ANOVA (FAD × SHRSP × sex) demonstrated a significant interaction between FAD and SHRSP. *Post hoc* analysis showed that Aqp-4 (150 kDa) was significantly elevated in plasma exosomes from SHRSP/FAD rats, compared to FAD and SHRSP rats (*p* < 0.05). Data represent means ± SEM. ^∗^*p* < 0.05, ^∗∗^*p* < 0.01, ^∗∗∗^*p* < 0.001.

Figure 6 shows staining forAqp-4, a membrane-bound protein in astrocyte end-feet on vessels that regulates water permeability. In WKY rats, Aqp-4 staining was associated with evenly distributed small tubular vessels (black arrows), while in FAD rats, there were patches of staining of vessels that appeared to be fragmented, tortuous and thin astrocyte processes (red arrows) and swollen vessels (blue asterisks). In SHRSP rats, there was an increase in staining of slightly distended but normally shaped tubular vessels (black arrows), compared to WKY.

The FAD-dependent increase in amorphous patches was exacerbated in SHRSP/FAD rats (red arrows). Two-way ANOVA showed no significant interaction of FAD × SHRSP effects for count and percentage area stained. *Post hoc* analysis showed that compared to FAD, there were more Aqp-4 puncta (*p* < 0.01) and higher percentage area stained (*p* < 0.01) in the hippocampus of SHRSP/FAD rats.

#### Aquaporin-4 Protein Levels Are Increased in the Hippocampus of FAD Rats

Lysates were prepared from hippocampal tissue, and levels of Aqp-4 were measured. Representative lanes of blots are shown in Figure 6B (right, mid panel). Since Aqp-4 is prone to multimeric aggregation, we used only 1 microgram of protein and did not boil the sample buffer. Three main bands were identified: 37, 50, and 150 kDa. Data from densitometric analysis were analyzed by two-way ANOVA (FAD × SHRSP × sex), which indicated a significant main effect of FAD on the 150 kDa Aqp-4 band. *Post hoc* analysis showed that total Aqp-4 was significantly elevated in FAD (*p* < 0.01) and SHRSP/FAD (*p* < 0.01), compared to WKY and SHRSP rats, respectively. There was also a sex effect, which *post hoc* analysis showed was due to higher Aqp-4 levels in females across groups. However, there were no differences between strains with sex. In conjunction with Aqp-4, GFAP was measured in the same blot after re-probing, and was found to positively correlate with levels of total (*r*^2^ = 0.379, *p* = 0.0003) or monomeric Aqp-4 (*r*^2^ = 0.216, *p* = 0.0097).

#### Aquaporin-4 Levels in Plasma Exosomes

Plasma exosomes were analyzed for Aqp-4 by western blot and relative optical density of the main band (150 kDa), was calculated (Figure 6C). The only significant effect observed in the two-way ANOVA was an interaction between FAD and SHRSP, which *post hoc* analysis showed was due to a robust increase in Aqp-4 in SHRSP/FAD rats that was higher than levels in FAD and SHRSP rats (*p* < 0.05).

### Collagen IV and PECAM-1 Are Elevated in Brains of SHRSP/FAD Rats

#### Collagen IV Immunohistochemistry

**FIGURE 7:**
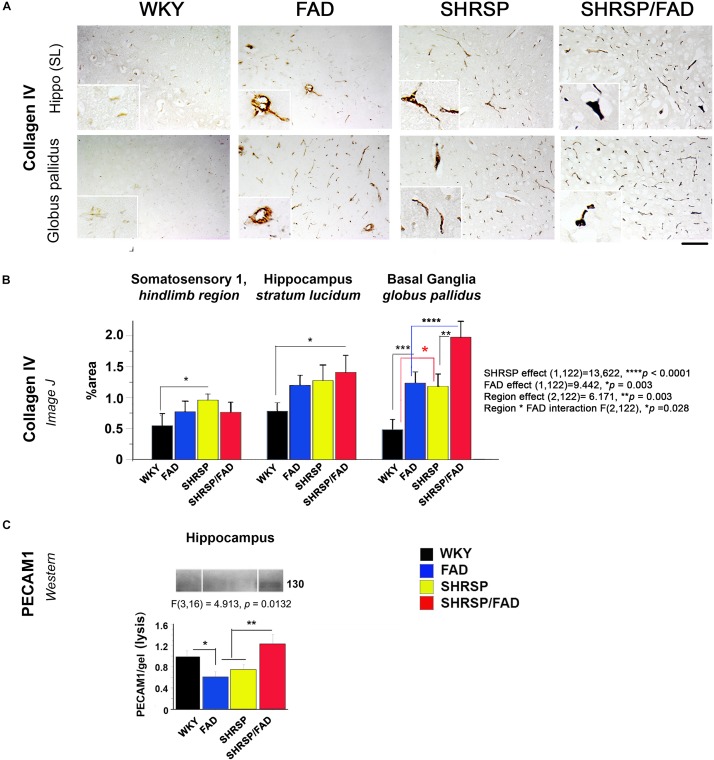
Collagen IV deposition is increased in FAD and SHRSP rats, while PECAM-1 is increased in SHRSP/FAD rats only. **(A)** Micrographs depict collagen IV staining in stratum lucidum of the hippocampus and globus pallidus of the basal ganglia. In all brain regions of WKY rats, vessels were lightly stained, and there was no staining in the vessel lumen. However, in SHRSP rats, the lumen of capillaries showed sparse collagen IV immunoreactivity. There was increased staining in the globus pallidus of FAD rats, which was exacerbated in SHRSP/FAD rats, in which vessel walls appeared distorted and thickened. **(B)** Two-way ANOVA was performed [region (three brain regions) × FAD (Tg+ or Tg–) × SHRSP (non-SHRSP or WKY)]. The main effects for both SHRSP were highly significant (*p* < 0.0001), and the effects for FAD, the effects of region and the interaction between FAD and region were also significant (*p* = 0.003, *p* = 0.003, and *p* = 0.028 respectively). *Post hoc* analysis with planned comparisons showed elevated intensity of collagen-IV staining in the hindlimb of SHRSP rats, compared to WKY (*p* < 0.05). In stratum lucidum of the hippocampus, collagen-IV staining was significantly elevated in SHRSP/FAD rats, compared to WKY (*p* < 0.05). Collagen-IV staining was increased significantly in the globus pallidus of FAD (*p* < 0.001) and SHRSP rats (*p* < 0.0001), compared to WKY. In SHRSP/FAD rats, collagen-IV staining was significantly increased further compared to FAD (*p* < 0.001) and SHRSP (*p* < 0.01) rats. **(C)** We also measured endothelial cell marker PECAM-1 in the lysis fraction of hippocampal tissue. Representative gel blots are shown and densitometry of bands was evaluated via one-way ANOVA, which demonstrated significant effects of strain on PECAM-1 levels. *Post hoc* analysis showed that levels of PECAM-1 were significantly elevated in the hippocampal lysis fraction from SHRSP/FAD rats, compared with FAD and SHRSP rats (*p* < 0.01). Levels of PECAM-1 were also significantly reduced in FAD rats, compared to WKY (*p* < 0.05). Data represent means ± SEM. ^∗^*p* < 0.05, ^∗∗^*p* < 0.01, ^∗∗∗^*p* < 0.001, ^****^p < 0.0001.

Figure 7 depicts collagen IV staining in the brains of the four strains, specifically the stratum lucidum of the hippocampus and the globus pallidus of the basal ganglia. In all brain regions of WKY rats, vessels were lightly stained with no collagen IV staining in the vessel lumen. However, the lumen of capillaries in SHRSP rats showed sparse collagen IV immunoreactivity. Within the globus pallidus, a noticeable increase in collagen IV staining in FAD rats was observed, which was further increased in SHRSP/FAD rats, where vessel walls appeared distorted and thickened. Two-way ANOVA of percentage area of collagen IV staining was performed (region × FAD × SHRSP) (Figure 7B). Main SHRSP (*p* < 0.0001) and FAD (*p* < 0.003) effects on percentage area stained for collagen IV were significant as for region (*p* = 0.003) and the interaction between region and FAD (*p* = 0.028), likely reflecting regional differences in the percentage area stained for collagen IV (the hindlimb of somatosensory cortex showed the least staining) and the more pronounced group effects in the globus pallidus. The hindlimb of somatosensory 1 was minimally affected: compared to WKY rats, only the percentage area of collagen IV staining was significantly increased in SHRSP (*p* < 0.05). In the hippocampus (stratum lucidum), the SHRSP/FAD group showed the highest percentage area stained (*p* < 0.05). In contrast to these modest effects, there were several group differences within the globus pallidus. Compared to WKY, collagen IV staining was significantly elevated in FAD (*p* < 0.001) and SHRSP (*p* < 0.05) and SHRSP/FAD (*p* < 0.0001) groups, and percentage area stained was higher in the SHRSP/FAD group than in all other groups (*p* < 0.0001).

#### PECAM-1 (CD31) Is Elevated in Brains of SHRSP/FAD Rats

By western blot, we assessed changes in the endothelial cell tight junction marker PECAM-1 in the cytoskeletal/lysis fraction, to where PECAM-1 is redistributed in response to chronic neuroinflammation and associated with disruption of the BBB (Romer et al., 1995; Ferrero et al., 1996). We measured PECAM-1 by western blot in the lysis fraction from hippocampal tissue as a biomarker of BBB leakage (Figure 7C). Representative gel blots are shown in the lower panel (Figure 7C). Densitometric analysis of bands was performed and one-way ANOVA demonstrated a significant effect of strain on PECAM-1 levels. *Post hoc* analysis showed that levels of PECAM-1 were significantly elevated in the hippocampal lysis fraction from SHRSP/FAD rats, compared with FAD and SHRSP groups combined (*p* < 0.01). Furthermore, PECAM-1 protein was significantly lower in hippocampal tissue from FAD rats, compared to WKY (*p* < 0.05).

### Calbindin Staining Is Reduced Slightly in SHRSP and Robustly in SHRSP/FAD Rats, While Caspase-Cleaved Actin Fractin Is Increased in SHRSP/FAD Rats

#### Calbindin Staining in the Thalamus and Hypothalamus Is Reduced in SHRSP/FAD Rats

**FIGURE 8:**
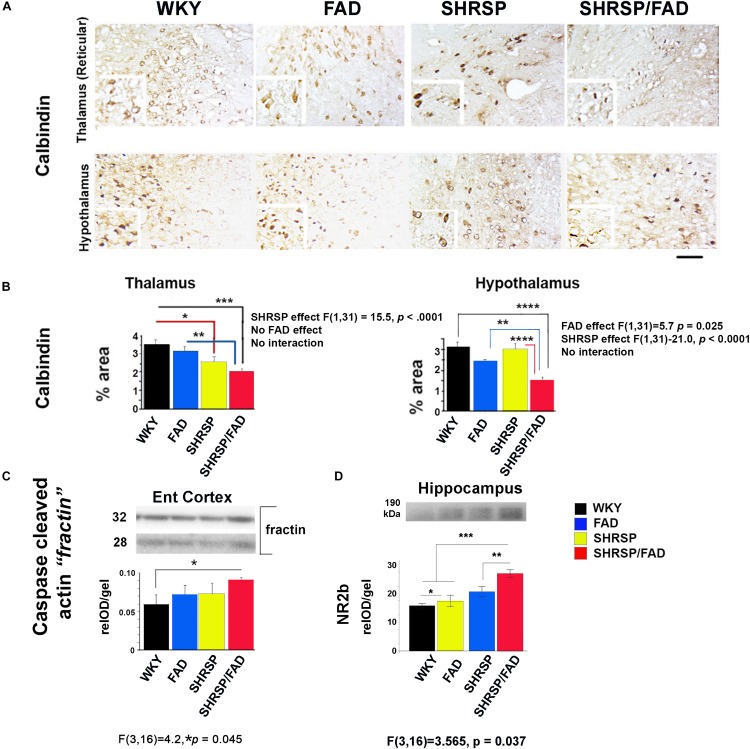
Neuron-specific calbindin is reduced in SHRSP/FAD rats, while caspase-cleaved actin is increased. **(A)** Micrographs depict calbindin staining in the hypothalamus and thalamus showing numerous calbindin-immunoreactive neurons. **(B)** Two-way ANOVA of percentage area stained (SHRSP × FAD) in the reticular thalamus showed no effect of sex, but a significant main effect of SHRSP (*p* < 0.0001), consistent with *post hoc* analysis, which showed that calbindin staining was reduced in SHRSP (*p* < 0.05) and SHRSP/FAD (*p* < 0.001) rats, compared to WKY. Furthermore, calbindin staining was significantly reduced in SHRSP/FAD rats, compared to FAD (*p* < 0.01), suggesting that FAD exacerbates neuron loss in SHRSP rats. Changes in calbindin staining in the hypothalamus were similar, and while there was a main SHRSP effect (*p* < 0.0001), there was an additional FAD main effect (*p* < 0.025). *Post hoc* analysis showed that calbindin staining was significantly reduced in the hypothalamus of SHRSP/FAD rats, compared to SHRSP (*p* < 0.0001), FAD (*p* < 0.01), and WKY (*p* < 0.0001) rats. **(C)** We also measured caspase-cleaved actin in the entorhinal cortex as an indication of caspase activity by western blot (lower panel). One-way ANOVA of relative optical density showed a significant strain effect (*p* = 0.045), and *post hoc* analysis showed that levels of caspase-cleaved actin were increased in SHRSP/FAD rats compared to WKY rats (*p* < 0.05). **(D)** Levels of post-synaptic NR2B were significantly different among strains. There were main effects of strain, and *post hoc* analysis showed a slight elevation of NR2B levels in FAD compared to WKY, but levels in SHRSP/FAD groups were higher than all other groups (*p* < 0.01). Data represent means ± SEM. ^∗^*p* < 0.05, ^∗∗^*p* < 0.01, ^∗∗∗^*p* < 0.001, ^****^p < 0.0001; one- and two-way ANOVA with LSD Fisher’s *post hoc* test.

To examine neuron damage we stained sections for calbindin, a neuroprotective calcium-binding peptide, which is predominantly enriched in GABAergic neurons, and its loss is associated with tangle pathology (Ahmadian et al., 2015). Images shown in Figure 8A illustrate positive calbindin staining in the reticular thalamus and the lateral hypothalamus. The intensity of staining of calbindin-immunoreactive neurons was variable across the strains, but the area stained was noticeably reduced in the SHRSP/FAD group. Two-way ANOVA of percentage area (SHRSP × FAD) in the thalamus showed a significant main effect of SHRSP, and *post hoc* analysis showed reduced calbindin levels in SHRSP (*p* < 0.05) and SHRSP/FAD (*p* < 0.001) rats, compared to WKY (Figure 8B). Furthermore, calbindin staining in the thalamus was significantly reduced in SHRSP/FAD rats, compared to FAD (*p* < 0.01), suggesting that calbindin-positive neuron loss in the thalamus was exacerbated in SHRSP/FAD rats, compared to FAD rats without hypertension. In the hypothalamus, there was a significant main effect of FAD (*p* < 0.025), in addition to a significant SHRSP effect (*p* < 0.0001). *Post hoc* analysis showed that percentage area of calbindin staining was significantly reduced in the hypothalamus of SHRSP/FAD rats, when compared to all other groups (*p* < 0.01) rats (Figure 8B). Together, these data support that the reduction of calbindin staining in the brains of SHRSP rats is exacerbated in SHRSP/FAD animals.

#### Levels of Most Synaptic Proteins Were Unchanged in the Hippocampus, While NR2B and Caspase-Cleaved Actin (Fractin) Were Elevated in SHRSP/FAD Rats

To further examine neurodegeneration, we measured synaptic proteins in membrane-enriched fractins in western blots (not shown). There were no changes in levels of pre-synaptic proteins SNAP25 and synaptophysin, nor in levels of the post-synaptic protein drebrin, however, levels of post-synaptic NR2B were different among strains (Figure 8D). *Post hoc* analysis showed that, although FAD showed a slight elevation in NR2B levels compared to WKY, NR2B levels were elevated in the SHRSP/FAD groups more than all other groups (*p* < 0.01).

We also measured caspase-cleaved actin (fractin (Yang et al., 1998), as an indicator of caspase-mediated apoptosis (Figure 8, lower panel). One-way analysis of relative optical density (relOD) of the bands showed a significant strain effect [*F*(3,16) = 4.2, *p* = 0.045] and *post hoc* analysis showed that levels of caspase-cleaved actin in the SHRSP/FAD rats were significantly higher than in WKY rats (*p* < 0.05).

### Luxol Fast Blue Staining Shows Demyelination in White Matter Tracts in SHRSP Which Is Exacerbated in SHRSP/FAD Rats

**FIGURE 9:**
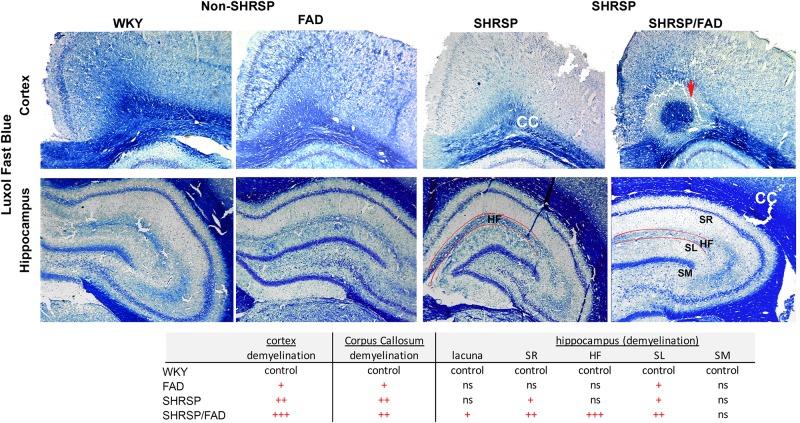
Demyelination in the cortex, corpus callosum and the hippocampal fissure of SHRSP/FAD rats. Sections were stained with Luxol fast blue to visualize changes in myelin distribution between the different strains. Qualitative evaluation by a blinded experimenter showed changes in myelin in both regions among the strains. Top panels show extensive, evenly spread Luxol fast blue staining, which densely labeled the corpus callosum (CC) of WKY rats. However, in FAD rats, there were dark patches of Luxol fast blue, interspersed with white patches of demyelination. In contrast to these minor changes in FAD rats, there was a notable loss of myelin patches in the CC and cortex of SHRSP and SHRSP/FAD groups. The top right panel shows a lacuna in the cortex-CC interface surrounded by a halo of demyelination (red arrow). There was no change in Luxol fast blue in the stratum molecular (SM) among strains, but in the FAD and SHRSP rats, there was some loss of myelin in the stratum radiatum (SR) and lucidum (SL) reflected by irregular patches of blue in the FAD rat, with more extensive loss in the SHRSP/FAD rats. The most pronounced loss of myelin was seen in the hippocampal fissure of SHRSP/FAD rats.

Figure 9 depicts representative images of the retrosplenial cortex and hippocampus of all strains stained with Luxol fast blue, to visualize changes in myelin distribution between strains. Qualitative evaluation by an experimenter blinded to transgene, showed changes in myelin in both regions among the strains. In the cortex and corpus callosum (CC) (top panel) of WKY rats, there was extensive Luxol fast blue staining, which densely labeled the CC and was evenly spread. However, in FAD rats there were dark patches of Luxol fast blue, interspersed with white patches of demyelination. In contrast to these minor changes in FAD rats, there was notable loss of myelin patches in both SHRSP groups in the CC and cortex. In the SHRSP/FAD rat occasional lacunae appeared in the perirhinal cortex (not shown) and in the cortex-CC interface surrounded by a halo of demyelination (top right panel).

Selective layers of the hippocampus were affected differentially in the four strains. There was no change in Luxol fast blue in the stratum molecular among strains, but in the FAD and SHRSP rats, there was some loss of myelin in the stratum radiatum and lucidum reflected in irregular patches of blue in the FAD rat brain, with more extensive loss in the SHRSP/FAD rats. The most pronounced loss of myelin was seen in the hippocampal fissure of the SHRSP/FAD rat, when compared with all other strains.

### Mitochondrial Complex I Is Reduced in Brains of SHRSP/FAD and FAD Rats, While Complex II Is Reduced in SHRSP/FAD Rats Only

**FIGURE 10:**
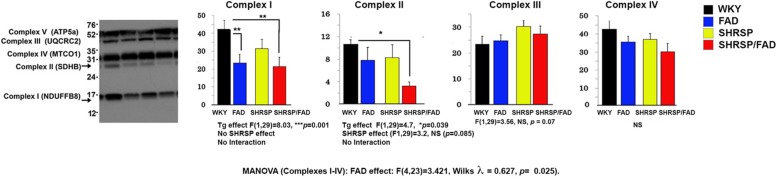
Mitochondrial complex II is reduced in the hippocampus of SHRSP/FAD rats and mitochondrial complex I is reduced in FAD rats. We measured mitochondrial complexes I-IV from hippocampal tissue by western blot and MANOVA was performed on the relative optical densities of protein bands from complexes I (log transformation), and II-IV. Results showed no sex effects, but a significant main FAD effect (*p* = 0.025) and the univariate test showed significant FAD main effects for Complexes I and II (*p* < 0.0001 and *p* < 0.039, respectively), but not III and IV. Although complex I proteins were significantly reduced in SHRSP/FAD (*p* < 0.01) and FAD (*p* < 0.01) rats, compared to WKY, complex II levels, were significantly reduced more than threefold in SHRSP/FAD rats, compared to WKY (*p* < 0.05). Data represent means ± SEM. ^∗^*p* < 0.05, ^∗∗^*p* < 0.01; MANOVA with Wilks λ test and LSD Fisher’s *post hoc* test.

Figure 10 shows representative lanes of the western blot of mitochondrial complexes I-V from hippocampal tissue. MANOVA was performed on protein band relOD of Complexes I (log transformation), and II -IV (Complexes I-IV × SHRSP × FAD). Results showed a significant main FAD effect [*F*(4,23) = 3.421, Wilks λ = 0.627, *p* = 0.025]. The univariate test showed significant FAD main effects on Complexes I and II (*p* < 0.0001 and *p* < 0.039, respectively), but not III or IV. Levels of Complex I were significantly reduced in FAD (*p* < 0.01) and SHRSP/FAD (*p* < 0.01) rats, compared to WKY, while Complex II was reduced three-fold in SHRSP/FAD rats only (*p* < 0.05). These data suggest that mitochondrial Complex I is depleted in the hippocampus of FAD rats only and Complex II is depleted in the SHRSP/FAD.

### SHRSP Does Not Exacerbate FAD Deficits in Y Maze and Novel Object Recognition but Has Independent Effect on Hyperactivity

#### Novel Object Recognition Task

Two-way ANOVA (object preference × strain) demonstrated a significant preference for the novel object during the test phase of the NOR task in WKY rats (*p* < 0.001), however, in FAD animals, the recognition index (RI) [(time exploring novel object)/(time exploring both objects)] for the novel object did not differ significantly from that of the familiar object (*p* > 0.05) in any of the other strains, indicating a failure to recognize the novel object as a result of impaired working memory in SHRSP, FAD and SHRSP/FAD rats. Similar results were observed when calculating a discrimination index (DI) [(novel time –familiar time)/(novel time + familiar time)].

**FIGURE 11:**
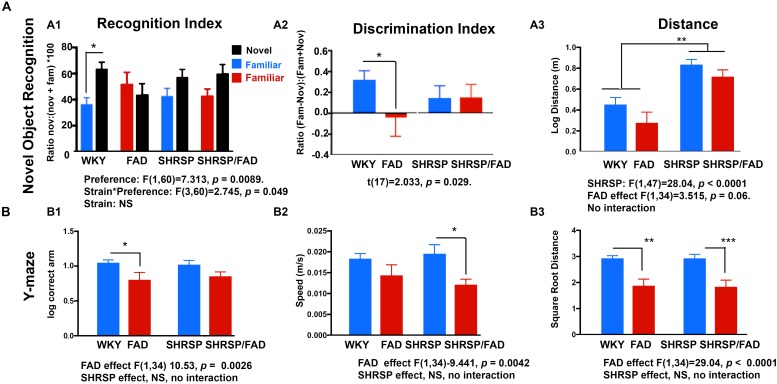
Performance in the novel object recognition and Y maze tasks by WKY, FAD, SHRSP and SHRSP/FAD rats. Data were analyzed by two-way ANOVA (FAD × SHRSP). **(A)** The novel object recognition (NOR) task involved 2 days of habituation, followed by exposure of the rats to two identical objects in an acquisition phase, prior to a test phase, following a retention period of 1 h. Graphs depict recognition indices (RI; time exploring novel or familiar object/time exploring both objects × 100) and discrimination index [DI; (time exploring novel object – familiar object)/(time exploring novel object + familiar object)] in the test phase of the NOR task in FAD (*n* = 11), SHRSP (*n* = 10), SHRSP/FAD (*n* = 9) and non-transgenic (*n* = 8) rats. Two-way ANOVA showed no sex effect, but there was an interaction between strain and object preference (*p* < 0.05) and object preference (*p* = 0.0089). Fisher’s LSD *post hoc* comparisons indicated that only WKY controls showed a significant preference for the novel object (*p* < 0.05). Similar results were seen with the two-way ANOVA with DI. Similarly, although two-way ANOVA showed that variation of the DI among groups in the test phase of the NOR task was not significant, *t*-tests within groups showed that the NOR test phase DI was significantly lower in FAD rats, compared to WKY (*p* = 0.029). However, the trends for lower DI in SHRSP and SHRSP/FAD rats, when compared with WKY, were non-significant. Two-way ANOVA of distance traveled during test phase of the NOR task showed a significant effect of SHRSP (*p* < 0.0001), and a trend for a main effect for FAD (*p* = 0.06). Fisher’s LSD *post hoc* analysis showed that distance traveled in the SHRSP group was significantly higher than in WKY (*p* < 0.01). Since there was no interaction, the near significance of FAD is attributed to similar reductions in distance, independent of SHRSP. **(B)** Performance in the Y maze is shown for FAD, SHRSP, SHRSP/FAD and WKY rats. No differences in spontaneous alternation were seen between strains (not shown). Correct arm entries were assessed by two-way ANOVA showing no interaction between strain and FAD, although there was a significant FAD effect (*p* < 0.0026). *Post hoc* analysis revealed that the number of correct arm entries was significantly lower in FAD rats only, compared to WKY (*p* < 0.05). Similarly there was a significant FAD effect on speed (*p* < 0.0042) and distance (*p* < 0.0001) in the Y maze. *Post hoc* analysis showed that speed was significantly lower in SHRSP/FAD rats, compared to SHRSP, while distance was reduced in FAD and SHRSP/FAD rats, compared to WKY (*p* < 0.01) and SHRSP (*p* < 0.001), respectively. Data represent means ± SEM. ^∗^*p* < 0.05, ^∗∗^*p* < 0.01, ^∗∗∗^*p* < 0.001; one-way ANOVA with Fisher’s LSD *post hoc* test.

It is noteworthy that distance (Figure 11A) and speed (not shown) were significantly higher in SHRSP and SHRSP/FAD animals during the NOR test phase, when compared with control and FAD rats (Figure 11). Similar hyperactivity was evident in SHRSP and SHRSP/FAD animals during the open field task (OFT) (not shown). Collectively these data are consistent with SHRSP not affecting preference for novel object with or without FAD, but causing hyperactivity independent of FAD.

#### Y Maze

During an 8-min Y maze task, correct arm entries, speed, distance (Figure 11B), spontaneous alternation, and errors (not shown) were assessed. Two-way ANOVA (FAD × SHRSP) showed significant main effects of FAD on correct arm entries, speed and distance (*p* < 0.005), but no SHRSP effects or interactions.

*Post hoc* analysis showed a correct arm entries were significantly lower in FAD rats compared to WKY (*p* = 0.0026), and while there was also a reduction in correct arm entries in the SHRSP/FAD rats, this difference did not reach significance. *Post hoc* analysis also showed that SHRSP/FAD rats were significantly slower in the Y maze than SHRSP rats (*p* = 0.0042), while a slight reduction of speed in FAD rats, compared to WKY, was not significant. Finally, FAD-dependent reductions in speed were shown by *post hoc* analysis, whereby FAD and SHRSP/FAD rats were significantly slower in the Y maze, when compared with WKY (*p* < 0.01) and SHRSP (*p* < 0.001) rats, respectively.

## Discussion

Here we present an initial characterization of a novel rat model of MxD, exhibiting complex interactions between chronic hypertension and AD pathology. Crossing the SHRSP background into the FAD rats (SHRSP/FAD) produces a line that retains both the AD phenotype with extensive plaque pathology as well as the SHRSP hypertensive phenotype. Importantly to facilitate the investigation of direct effects of hypertension on AD dementia, we created a FAD+ control line, by introducing WKY background (non-hypertensive background strain for the SHRSP), which retained the FAD transgene-dependent neuritic plaque phenotype and behavioral deficits of the original F344 line.

Our data show region-dependent synergistic interactions, with exacerbated metabolic deficits, demyelination, gliosis, tau pathology and neurodegeneration in SHRSP/FAD rats, which is consistent with clinical and neuropathological data suggesting that coexistence of subclinical AD and SVD pathologies may lower the threshold for dementia risk (Fischer et al., 1991). We also observed exacerbation of SHRSP-dependent vascular changes in SHRSP/FAD rats, indicative of leakage and disruption of the BBB, but without greatly exacerbating AD plaque burden, consistent with the clinical neuropathological data on neuritic plaques in MxD (Petrovitch et al., 2005). This novel model should allow a better understanding of how hypertension may differentially affect neuritic and diffuse plaque and vascular amyloid pathology. It may provide a useful model for translation to identify how hypertensive phenotypes may interfere with the efficacy of some AD therapeutics, possibly even when vascular or AD pathological markers are below levels of detectability with standard neuroimaging (Fischer et al., 1991; Baker et al., 2012). This type of model is critical as undetected vascular damage could limit the efficacy of drugs that target AD pathology alone. Moreover, this model may be useful for preclinical research aimed at delineating the pathophysiology of VaD and MxD and development of therapies for patients with MxD, in which the causes of cognitive decline are complex and therapeutic efficacy and safety may be limited by coexisting pathologies in MxD or VaD.

### Subtle Changes in Plaque Pathology in the SHRSP/FAD Rat

SHRSP did not have an overwhelming impact on overall plaque burden in the FAD rats, as it slightly reduced Aβ staining in entorhinal and perirhinal cortices of SHRSP/FAD rats, increased Aβ in the hilus and vessel-rich hippocampal fissure and altered plaque morphology, reducing the plaque’s neuritic dense-core plaques decorating FAD brain. This is contrast to the models with hypertension induced increases in Aβ discussed in the introduction. Although we lack rigorous clinical data describing differences in plaques between AD and MxD, our data is consistent with findings in the Honolulu Aging Study showing increased dementia risk developing in cognitively normal men with vascular pathology who were found to have increased tau pathology but fewer neuritic amyloid plaques (Petrovitch et al., 2005). Together these data support that, although hypertension exerts complex effects on Aβ pathology, it does not accelerate plaque pathogenesis in our model or in humans.

Whether or not hypertension attenuates the evolution of the diffuse plaque into a dense neuritic plaque or directly affects preexisting neuritic plaques, regional differences in neuroinflammatory responses could explain this effect. Compared to WKY, we observed relatively region-independent increased neuroinflammatory responses in SHRSP. FAD rats had much higher glial responses than SHRSP rats but this was region-dependent and particularly high in plaque-rich regions like the hilus, with disproportionate plaque burden, and less where there were fewer plaques (entorhinal, perirhinal), consistent with a strong inflammatory response to plaques. Therefore, the regional variations in plaques in SHRSP/FAD could be related regional glial responses to plaques, increasing or attenuating clearance. It should also be investigated whether diminished plaques could occur directly in response to hypertension as in the mutant APP crossed onto db/db mice which have hypertension and Type II diabetes and show a slight reduction in plaques (Ramos-Rodriguez et al., 2015; Infante-Garcia et al., 2016) or result secondary to enhanced clearance from lacunar stroke and increased monocyte/macrophage invasion (Frautschy et al., 1992).

In addition to possible innate immune clearance mechanisms, our model may impact LRP1 which mediates Aβ efflux in cerebral microvessels (Donahue et al., 2006; Kanekiyo et al., 2012) or receptor for advanced glycation end products (RAGE) in neurons which can mediate Aβ influx (Deane et al., 2003) or LDLR that can reduce Aβ (Kim et al., 2009; Katsouri and Georgopoulos, 2011). Specifically it would be important to examine redistribution of RAGE and LRP1 between microvasculature and neurons which appears Aβ or AD specific (Donahue et al., 2006; Shang et al., 2019) and increased expression of the low-density lipoprotein receptor (LDLR) which appears hypertension or SHRSP-specific (Ueno et al., 2010). However, whatever the mechanisms involved are, the Aβ effects are surprisingly small and regional and not the salient interactive feature in our model or in humans. The mechanisms determining potentially regional changes in Aβ clearance affecting plaque pathology include regional cerebral hypoperfusion and are clearly complex and poorly understood. Notably, two studies with surgically induced or angiotensin II infusion-induced hypertension did not show hypertension-associated amyloid reduction (Cifuentes et al., 2015; Shih et al., 2018). Although those studies did not evaluate plaque burden in the regions described here, they support our conclusions that the impact on plaques is subtle and requires more thorough examination of vascular amyloid and the relationship of vessels to focal changes in cerebral blood flow, neuroinflammation, lacunar or vascular changes, plaque size, texture and distribution in the SHRSP/FAD rat and in patients with MxD.

One limitation of our study is that because amyloid effects were small and regional, we did not pursue regional effects of SHRSP on amyloidogenic APP processing or changes in soluble oligomeric species. One might expect that there would be synergism between effects of hypertension and AD on APP processing, since hypertension models suggest that with aging hypertension increases the activity of β-secretase (not mRNA) as well as APP binding proteins (Csiszar et al., 2013; Zhang et al., 2018, 2019) and in AD models, expression of β-site amyloid precursor protein-cleaving enzyme 1 (BACE1) can be upregulated by oxidative damage or inflammation (Zhang et al., 2018). However, synergism between effects of hypertension and AD on APP processing as a primary mechanism, is not supported by the subtle differences in Aβ observed in our model and in human MxD data.

Diffuse extracellular Aβ has been reported in brains of SHRSP rats (Bueche et al., 2014; Schreiber et al., 2014) using anti-Rodent Aβ antibody, which binds to the three rodent specific amino acid differences present in Aβ 1-16, but also in full length APP and its C-terminal fragment. Unfortunately, labeling with this class of 1–16 region antibody also detects APP and its non-Aβ products associated with sites of axonal injury as well as Aβ. While we used polyclonal anti human Aβ (Launer et al., 2000; Petrovitch et al., 2000; Ferri et al., 2005; Petrovitch et al., 2005; Jellinger and Attems, 2007; Kalaria et al., 2012; Hebert et al., 2013; Rodrigue et al., 2013; Wimo et al., 2013; Gottesman et al., 2014; Oberlin et al., 2015; Khan et al., 2016; Custodio et al., 2017; Walker et al., 2017; Vinters et al., 2018; Wennberg et al., 2019) and showed that SHRSP rats showed rare sparse vascular Aβ pathology and rare diffuse plaques, our findings are still in agreement with Schreiber’s group who found limited Aβ deposition or neuritic plaques in SHRSP rats in contrast to the extensive deposition observed in our FAD and SHRSP/FAD groups.

### Robust Changes in Tau Pathology in the SHRSP/FAD Rat

The F344 Tg AD rat, from which our model was derived exhibits amyloid plaque pathology, insoluble tau and age-dependent cognitive deterioration, but surprisingly insoluble tau is reduced with age in the original report (Cohen et al., 2013). However, the introduction of hypertension in SHRSP/FAD rats robustly increased tau pathology. Importantly these data show that low levels of vascular pathology caused by hypertension may exacerbate tau pathology, despite minimal effects on amyloid pathology. There has not been research directly investigating hypertension and tau, however, there are two reports that indirectly support our observations. First, APP mice with the hypertensive db/db background show increased phospho- tau levels (Ramos-Rodriguez et al., 2015; Infante-Garcia et al., 2016), but increased Aβ and neuroinflammation associated with diabetes in this model could have driven tau hyperphosphorylation independent of hypertension. As described above, cognitively normal men with hypertension, who have fewer neuritic plaques but more tau pathology are at increased risk for AD (Petrovitch et al., 2005). Thus, if validated with further clinical and longitudinal neuropathological studies, our initial finding that hypertension increases tau pathogenesis in our MxD model would be an important advance in the field.

The Schreiber group reported that the SHRSP rat exhibits tau hyperphosphorylation (Schreiber et al., 2014), however, no evidence of unambiguous neurofibrillary tangles or detergent insoluble tau was established. Because hyperphosphorylation of tau is commonly observed with rodent brain injury and inflammation in the absence of detergent insoluble tau or tangles, findings of increased ptau by ICC or soluble ptau cannot be conflated with insoluble aggregating tau or tangles but it may indicate a relevant imbalance in tau kinases, phosphatases or clearance mechanisms. While p-tau levels in the present study were comparable between FAD and SHRSP rats, contrary to (Schreiber et al., 2014), several differences exist between their methods and ours. (1) the Schreiber laboratory used AT8 [to both Tau pSer202 and pThr205 (Schreiber et al., 2014) while we used pS422 and CP13 to Tau pSer422 and Tau pSer202, respectively; (2) they did not used phosphatase inhibitors that prevent post mortem phosphorylation, (3) they only used males which have more hypertension; and (4) they evaluated the cortex while we evaluated hippocampus. Nevertheless, our combined results suggest chronic hypertension may promote tauopathy, a known path to neurodegeneration.

### Robust Increases in Microglial and Astrocytic Neuroinflammation in the SHRSP/FAD Rat

Similar to AD (Akiyama et al., 2000) and CBVD (Gallart-Palau et al., 2017), there was increased microgliosis and hyperplasia of astrocytes in both FAD and SHRSP rats. Although increased neuroinflammation has previously been reported in both models (Sabbatini et al., 2002; Cohen et al., 2013; Tayebati et al., 2016), this is the first observation showing an additive response with the coexistence of robust AD pathology and hypertension. Specifically, brains of SHRSP/FAD rats showed increased glial density and percentage of positive staining. Notably, average microglia and astrocyte cell size were further elevated in the cross, indicative of reactive cells. Compared with SHRSP rats, which are not reported to show hypertrophy of astrocytes (Sabbatini et al., 2002; Tayebati et al., 2015), astrocytes in brains of SHRSP/FAD rats were significantly larger. Our data suggest that the combination of SHRSP phenotype with the AD transgene in the SHRSP/FAD rat enhances gliosis and possibly neuroinflammation generally in the brain beyond that seen with either alone which suggests a synergistic amplification of this pathology caused by the coexistence of AD pathology and hypertension. Further examination of the transcriptomic and functional profiles of glial cells in specific regions of these animals will help to clarify the effect that hypertension has on glial cells in the context of AD. Not only was microgliosis increased in SHRSP/FAD rats, microglial morphology was also different, compared with all other animals. Many microglia in the brains of SHRSP/FAD rats were rod-shaped and had more processes, such that there was more total Iba1-positive area stained, despite fewer microglial cells. Notably, several microglia had larger cell bodies, which typically associated with microvessels. Previously in a mouse model of stroke, it was found that microglia played a pathogenic role surrounding and phagocytosing endothelial cells leading to the disintegration of vessels, which was in part stimulated by fibrinogen or albumin, both indices of vessel leakage (Jolivel et al., 2015). While we did observe vessel-associated microglia and the density of vessels associated with Aqp-4 and collagen IV, we did not evaluate the density of all vessels. However, dysregulated Aqp-4 staining, an astrocytic endfoot protein that associates with cerebral blood vessels, in the SHRSP/FAD rat is consistent with damage to the neurovascular unit.

### Aquaporin-4 in Microvessels in the SHRSP/FAD Rat

Parallel to GFAP changes, the astrocytic endfoot protein Aqp-4 was upregulated in the hippocampus of SHRSP/FAD rats. It is known that Aqp-4 is increased in brains of SHRSP rats (Tomassoni et al., 2010; Tayebati et al., 2015) and in AD brain, in association with amyloid plaques (Hoshi et al., 2012; Yang et al., 2017). Our data indicate that this upregulation of Aqp-4 is amplified in the SHRSP/FAD rat. A positive correlation between Aqp-4 and GFAP protein levels are consistent with prior reports of increased Aqp-4 corresponding with increased gliosis in a model of hypoglycemia (Zhao et al., 2018). Aqp-4 staining was redistributed to the neuropil, but it was unclear whether the loss of polarization was due to disintegration of vessels or retraction of endfeet. Dysregulation of Aqp-4 may be associated with cognitive dysfunction and pathology in AD and VaD (Lan et al., 2017). In fact in chronic traumatic encephalopathy (CTE), Aqp-4 is also increased and associated with perivascular tau (McKee and Robinson, 2014; Babcock, 2018) or age-related tau astrogliopathy (Kovacs et al., 2017). This similarity supports the concept the chronic damage to the neurovascular unit may contribute to tauopathy in both CTE and MxD.

### Aquaporin-4 as a Blood Biomarker

Not only was there disruption of Aqp-4 distribution in the brain, elevated levels of Aqp-4 protein were additionally detected in brain-derived plasma exosomes from SHRSP/FAD rats. As such, Aqp-4 may prove to be a useful peripheral blood biomarker of MxD or VaD, reflecting vascular and inflammatory changes in the brain as a result of AD pathology with concomitant vascular dysfunction, thereby allowing for stratification of patients with a more complex dementia and guiding potential treatment options. In fact, others have shown in humans that neuron-derived plasma exosomes from traumatic brain injury (TBI) patients contain elevated levels of Aqp-4 protein, compared to controls and that Aqp-4 levels were significantly higher in exosomes from acute TBI patients, compared to chronic (Goetzl et al., 2019), suggesting that Aqp-4 levels in plasma exosomes may represent a robust marker with which to stratify patient groups or identify a clinically silent traumatic or indeed neurodegenerative brain disorder. Whether the findings in our model are relevant to serum changes in VaD or MxD patient samples should be examined.

### Evidence of Loss of Microvessel Integrity

We observed excessive accumulation of collagen IV in brains of SHRSP/FAD rats, which may reflect the development of cerebral SVD, which is aggravated by AD pathology. Chronic hypertension increases collagen deposition in cerebral vessel walls (Zhou et al., 2015) and leads to deficient vessel integrity and function (Feihl et al., 2008; Grinberg and Thal, 2010; Heagerty et al., 2010). Similarly dysfunctional vessels are evident in brains of AD patients (Farkas et al., 2000; Bouras et al., 2006; Kitaguchi et al., 2007), and other hypertensive models with AD showed reduced vessel density (Cifuentes et al., 2015) or leakage (Shih et al., 2018). Interestingly, we found that SHRSP/FAD rats also showed elevations in the endothelial cell adhesion protein platelet endothelial cell adhesion molecule-1 (PECAM-1), which is involved with trans-endothelial leukocyte and monocyte migration across into the brain as part of the neuroinflammatory response (Giri et al., 2000, 2002; Kalinowska and Losy, 2006). Elevated PECAM-1 also implies that vascular inflammatory signaling at least plays a role in driving neuroinflammation in the brain of SHRSP/FAD rats. Our data show that long-term mild hypertension is associated with abnormal accumulation of vascular collagen IV, altered PECAM-1 and disruption of astrocyte end-feet associated with vessels. These findings suggest that AD pathology with concurrent chronic hypertension exacerbates a loss of CNS blood vessel integrity.

### Mitochondrial Deficits Unique to the SHRSP/FAD Rat

Recent work has probed the involvement of dysfunctional mitochondria and bioenergetic deficits in AD pathophysiology. Mitochondrial dysfunction is an early event in AD pathogenesis (Yao et al., 2009; Calkins et al., 2011; Varghese et al., 2011; Du et al., 2012) leading to ATP depletion, which ultimately contributes to synapse and neuron degeneration (Vos et al., 2010; Swerdlow, 2018). In FAD rats, mitochondrial complex I (NADH:ubiquinone oxidoreductase) but not complex II (succinate dehydrogenase) was downregulated. The FAD effects on complex I are consistent with findings in AD patients, in which the 24 or 75 kDa subunits were reduced in the parietal, occipital and temporal cortices and caudate nucleus (Kim et al., 2001). This suggests that AD may have an early effect on mitochondrial complex I.

The FAD rats showed no changes in complex II consistent with reports that AD patients show no changes or even increased complex II protein expression (Bubber et al., 2005). Apparent increases in complex II specific to AD have been attributed to compensatory responses facilitating anaerobic metabolism as a result of loss of complexes, such as pyruvate dehydrogenase, involved in oxidative metabolism. Importantly SHRSP/FAD rats instead showed reductions in complex II, which would be predicted to compound bioenergetic deficits. Differences in complex II in MxD versus AD have not been looked at to the best of our knowledge, other than one report that found no changes in isolated VaD. However, complex II was found to be reduced in a rat model of VaD (Singh et al., 2015) and in a traumatic brain injury model in brain regions with disrupted cerebral blood flow (Jiang et al., 2000). Together these data warrant a more thorough examination of differential mitochondrial deficits in VaD, AD and MxD to determine if additional mitochondrial and fluorodeoxyglucose (FDG)-positron emission tomography (PET) deficits are promoted by the coexistence of both pathologies.

### Evidence for Neuronal Damage in the SHRSP/FAD

Hypertension appeared to amplify neurodegenerative responses in AD rats. Here we showed that calbindin staining was reduced in brains of hypertensive SHRSP/FAD animals, possibly a reflection of increased neuronal vulnerability or loss as a result of the combination of hypertension with AD pathology. The FAD rats utilized in this study exhibit stereology-validated neuronal loss (Cohen et al., 2013), however, the SHRSP/FAD rats exhibited further reductions of calbindin, suggesting that neuron or calbindin loss might be intensified in the cross. Furthermore, caspase-cleaved actin levels were elevated in SHRSP/FAD brain, which had previously been reported in AD (Yang et al., 1998). Increased caspase activity may also relate to the hyper-inflammatory environment of the brain in SHRSP/FAD rats, through its interaction with the NOD-, LRR- and pyrin domain-containing protein 3 (NLRP3) inflammasome, a known hub of pathology in AD brain that drives interleukin-1β (IL-1β) production via TLR4 signaling (Burm et al., 2015). Although not investigated in this initial report, it will be important for future studies to assess inflammatory receptor signaling and cytokine profiles in the brain of SHRSP/FAD rats and potential correlations with tau hyperphosphorylation, tauopathy and neuron loss. Reduced brain volumes, including for the hippocampus and temporal lobes, have been found in individuals with elevated blood pressures, recorded several years prior (Beauchet et al., 2013; Power et al., 2016) and in animals in which hypertension was induced (Meissner et al., 2017). This may be due to neuron and pericyte loss as a result of chronic hypertension independent of tauopathy (Goel et al., 2015; Kruyer et al., 2015). Thus, our caspase and calbindin data add support for an amplification of neurodegenerative responses caused by the coexistence of both pathologies in SHRSP/FAD rats.

### Pathological Elevations of NR2B in the SHRSP/FAD Rat

We observed significant increases in hippocampal NR2B protein detected by western blot in SHRSP/FAD rats. NR2B is a subunit of the *N*-methyl-D-aspartate receptor (NMDAR), an ionotropic glutamate receptor expressed at the post-synaptic membrane that can enhance memory (Cao et al., 2007; Wang et al., 2009; Vedder et al., 2013; Wang, 2014). Although AD typically shows disproportionate loss of post synaptic proteins, synaptic marker loss is both stage and region dependent as there can be compensatory sprouting which could be either pathological (aberrant sprouting) or transiently protective and an influx of aberrant terminals around plaques. Pathological elevations have been described associated with apoptosis in AD models (Liu et al., 2012). While overactivation of NR2B-containing NMDARs might improve some aspects of hippocampal-dependent memory, it can also contribute to excitotoxic calcium flux, hyperactivity and to the accumulation of hyperphosphorylated tau in the hippocampus following ischemic injury through disinhibition of glycogen synthase kinase-3β (GSK-3β), an important tau kinase (Xu et al., 2015). The elevated Tau pS422 staining and CP13 protein detected in our hypertensive AD rats is likely due to several factors, including neuroinflammation which has been shown to drive tau pathogenesis in AD models through effects on cytokine-induced tau hyperphosphorylation (Ghosh et al., 2013). Under normal conditions, neurons, but not astrocytes, express NMDARs; but following ischemic insult, expression of NMDARs is also evident in astrocytes (Krebs et al., 2003). Detailed longitudinal immunohistochemical studies are required to examine the regional changes in NR2B and phospho-NR2B and their possible relationship with cognitive performance.

### Behavioral Changes in FAD and SHRSP Rats Were Not Exacerbated in SHRSP/FAD Rats

Limited behavioral testing was performed, and we found that independent effects of FAD and SHRSP were unaffected in SHRSP/FAD by the coexistence of both features and prominently impacted by hyperactivity in SHRSP. In particular, hypoactivity in the Y maze was dependent on FAD and independent of SHRSP, while hyperactivity in both the OFT and NOR was dependent on SHRSP and independent of FAD. Neuropsychologically, little is understood about synergism of AD and vascular pathology in MxD as the current criteria for MxD would require fitting the diagnosis of both AD and VaD. For example one would expect declarative memory deficits related to AD and executive dysfunction relative to VaD, and predominantly with MxD this would be occurring in the older population (averaging 83 years old) (Custodio et al., 2017). It is speculated that the coexistence of both pathologies would lower the threshold of dementia, whereby less AD and less vascular pathology could cause dementia, even if neither pathology would by itself fulfill the pathological criteria for one or the other (Fischer et al., 1991).

There was a ceiling effect in NOR deficits, such that WKY rats, but none of the other groups showed preference. Thus, we could not evaluate whether the presence of hypertension affected severity of FAD deficits using the NOR task.

Limitations in the type of behavioral testing included not being able to complete more complex tests such as water maze or Barnes maze, that have been used to detect cognitive impairment in these models. We did, however, detect SHRSP-dependent hyperactivity, which is a recognized phenotype in this model (Gattu et al., 1997a, b; Terry et al., 2000; Hernandez et al., 2003; Kantak et al., 2008; Meneses et al., 2011). Since patients with MxD are typically older, another limitation in our study is that we do not have data on progression, so we do not know if there would be exacerbation of pathology with older ages or with strokes which we can but did not enhance with higher salt intake in our initial characterization. Therefore, although we identified independent behavioral changes associated with FAD transgene or SHRSP background with the minimal testing done, it is important to test these rats in the future with other behavioral tasks to determine which type of behaviors are exacerbated by the coexistence of both hypertension and FAD with and without higher salt intake. Future studies should focus on behavioral tests analogous to known differences in neuropsychological characteristics between MxD, VaD and AD such as behavioral testing in animals that reflects differences in episodic memory and executive function. Since VaD is associated with more anxiety and depression (reviewed by Cerejeira et al., 2012) we were not surprised to observe independent effects of AD and SHRSP, but we were surprised not to see more enhanced baseline cognitive deficits. For future studies we will explore whether a more comprehensive neuropsychological battery, assessing rat declarative memory (Engelmann et al., 2011) and executive dysfunction (Beas et al., 2013) or high salt challenge may reveal synergistic deficits caused by the hypertensive and FAD phenotypes. Finally, in tests where SHRSP show improved learning in young animals, a longitudinal evaluation of the age-related decline within strains rather than comparison of performance across strains will provide a better measure of the relationship of age-dependent pathology with age-dependent cognitive decline.

## Conclusion

In conclusion, we show here that this novel rat model of MxD exhibits robust neuropathology, including amyloid and tau pathology, gliosis and behavioral alterations. In addition, exacerbation of several disease parameters was noted in SHRSP/FAD, compared to SHRSP and FAD rats, including increased astrocytosis, Aqp-4, collagen IV deposition, PECAM-1, caspase activity and qualitatively increased demyelination with reduced mitochondrial enzymes and calbindin levels. This novel model appears to be an advancement in mixed models of dementia, which have relied primarily on major vessel occlusion or mouse models of diabetes and AD that express Aβ but not tau pathology. Our model more accurately reflects pathology in the human syndrome of MxD associated with chronic hypertension, in the absence of diabetes, developing alongside classical amyloidogenic pathology, in addition to tauopathy and neuron loss. Further work is needed to characterize more completely any cognitive or behavioral abnormalities in the SHRSP/FAD rat and also to determine the extent to which degeneration of neurons and synapses develops in this rat with and without salt challenge and small strokes, another hallmark of MxD. We believe that this novel disease model will support researchers in delineating the association between cardiovascular abnormalities and dementia and will help bolster efforts to develop treatments for AD, VaD and CBVD.

## Data Availability Statement

All datasets generated for this study are included in the manuscript/supplementary files.

## Ethics Statement

All experimentation was approved by the UCLA Chancellor’s Animal Research Committee and the Veteran Administration Institutional Animal Care and Use Committee, and carried out in compliance with the National Institutes of Health “Guide for the Care and Use of Laboratory Animals” (NIH Publications No. 8023).

## Author Contributions

SF designed and supervised the experiments, and assisted in manuscript writing, figures preparation, and statistical analysis. PD assisted in manuscript writing, and behavioral testing and analysis. SH performed and analyzed the Western blot. MJ performed and analyzed the immunohistochemistry. HD’A assisted in writing the background and assessment of clinical aspects of the disease. HV assisted in interpretation of the plaque morphology. CO performed the metabolic studies. PK and DC assisted in creation of the model, behavioral testing, animal euthanasia, and tissue preparation. CL and MM assisted in behavioral testing, and tissue preparation and sectioning. CZ performed the blood pressure measurement. EG assisted in writing background. BT assisted in blood pressure measurements. GC assisted in concepts for the study, and writing and editing of the manuscript. XZ prepared and analyzed the plasma exosomes.

## Conflict of Interest

The authors declare that the research was conducted in the absence of any commercial or financial relationships that could be construed as a potential conflict of interest.
